# Succinate receptor 1 signaling mutually depends on subcellular localization and cellular metabolism

**DOI:** 10.1111/febs.17407

**Published:** 2025-01-21

**Authors:** Aenne‐Dorothea Liebing, Philipp Rabe, Petra Krumbholz, Christian Zieschang, Franziska Bischof, Angela Schulz, Susan Billig, Claudia Birkemeyer, Thanigaimalai Pillaiyar, Mikel Garcia‐Marcos, Robert Kraft, Claudia Stäubert

**Affiliations:** ^1^ Rudolf Schönheimer Institute of Biochemistry, Medical Faculty Leipzig University Germany; ^2^ Research Group of Mass Spectrometry, Institute of Analytical Chemistry Leipzig University Germany; ^3^ German Center for Integrative Biodiversity Research (iDiv) Halle‐Leipzig‐Jena Germany; ^4^ Institute of Pharmacy, Pharmaceutical/Medicinal Chemistry and Tübingen Center for Academic Drug Discovery Eberhard Karls University Tübingen Germany; ^5^ Department of Biochemistry & Cell Biology, Chobanian & Avedisian School of Medicine Boston University MA USA; ^6^ Department of Biology Boston University College of Arts & Sciences MA USA; ^7^ Carl Ludwig Institute for Physiology, Medical Faculty Leipzig University Germany

**Keywords:** SUCNR1, succinate, macrophages, metabolism, signal transduction

## Abstract

Succinate is a pivotal tricarboxylic acid cycle metabolite but also specifically activates the G_i_‐ and G_q_‐coupled succinate receptor 1 (SUCNR1). Contradictory roles of succinate and succinate‐SUCNR1 signaling include reports about its anti‐ or pro‐inflammatory effects. The link between cellular metabolism and localization‐dependent SUCNR1 signaling qualifies as a potential cause for the reported conflicts. To systematically address this connection, we used a diverse set of methods, including several bioluminescence resonance energy transfer‐based biosensors, dynamic mass redistribution measurements, second messenger and kinase phosphorylation assays, calcium imaging, and metabolic analyses. Different cellular metabolic states were mimicked using glucose (Glc) or glutamine (Gln) as available energy substrates to provoke differential endogenous succinate (SUC) production. We show that SUCNR1 signaling, localization, and metabolism are mutually dependent, with SUCNR1 showing distinct spatial and energy substrate‐dependent G_i_ and G_q_ protein activation. We found that Gln‐consumption associated with a higher rate of oxidative phosphorylation causes increased extracellular SUC concentrations, accompanied by a higher rate of SUCNR1 internalization, reduced miniG_q_ protein recruitment to the plasma membrane, and lower Ca^2+^ signals. In Glc, under basal conditions, SUCNR1 causes stronger G_q_ than G_i_ protein activation, while the opposite is true upon stimulation with an agonist. In addition, SUCNR1 specifically interacts with miniG proteins in endosomal compartments. In THP‐1 cells, polarized to M2‐like macrophages, endogenous SUCNR1‐mediated G_i_ signaling stimulates glycolysis, while G_q_ signaling inhibits the glycolytic rate. Our results suggest that the metabolic context determines spatially dependent SUCNR1 signaling, which in turn modulates cellular energy homeostasis and mediates adaptations to changes in SUC concentrations.

## Graphical abstract

SUCNR1, activated by succinate, is a G_i_‐ and G_q_‐coupled receptor present at the plasma membrane and in endosomes. There are conflicting reports about the pro‐/anti‐inflammatory effects of the succinate‐SUCNR1 signaling axis, and these are likely due to localization‐dependent signaling. Here, we show that SUCNR1 signaling, localization, and metabolism are mutually dependent, and that M2‐like macrophages express SUCNR1. SUCNR1‐mediated G_i_ protein‐coupling stimulates oxidative phosphorylation and glycolytic rate and activates ERK and Akt phosphorylation. Conversely, Akt phosphorylation and glycolytic rate (extracellular acidification rate) are inhibited by SUCNR1 through G_q_ protein activation. Created with Biorender.com.

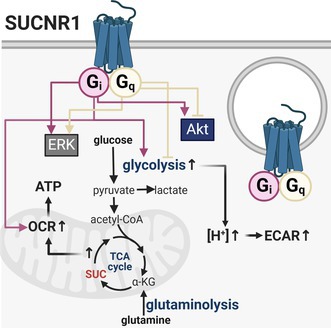

**Abbreviations**
ATP: adenosine triphosphate
BRET: bioluminescence resonance energy transfer
cAMP: cyclic adenosine monophosphate
CES: *cis*‐epoxysuccinate
CTRL: control
CCL: C–C motif chemokine ligand
CXCL: C‐X‐C motif chemokine ligand
DAG: diacylglycerol
DMEM: Dulbecco's modified Eagle medium
DMR: dynamic mass redistribution
DMSO: dimethylsufoxide
ECAR: extracellular acidification rate
ELISA: enzyme‐linked immunosorbent assay
ETC: electron transport chain
FCCP: carbonyl cyanide‐*p*‐trifluoromethoxyphenylhydrazone
FN1: fibronectin
GC–MS: gas chromatography–mass spectrometry
Glc: glucose
Gln: glutamine
GLS1: phosphate‐activated mitochondrial glutaminase
GPCR: G protein‐coupled receptor
HA: hemagglutinin
HATU: *O*‐(7‐azabenzotriazol‐1‐yl)‐*N*,*N*,*N′*,*N′*‐tetramethyluronium hexafluorophosphate
HBSS: Hank's balanced salt solution
HLA‐DRA: major histocompatibility complex, class II, DRα
IL‐1β: interleukin‐1β
IL‐10: interleukin‐10
IP_1_
: inositol‐1‐phosphate
mas: membrane anchoring sequence
MCT: monocarboxylate transporter
Nluc: nanoluciferase
NMOC: non‐mitochondrial oxygen consumption
OXPHOS: oxidative phosphorylation
pm: picometer
PTX: Pertussis toxin
ROS: reactive oxygen species
RPMI: Roswell Park Memorial Institute
SDH: succinate dehydrogenase
SLC13: solute carrier family 13
SUC: succinate
SUCNR1: succinate receptor 1
TCA: tricarboxylic acid
THP‐1: Tohoku hospital pediatrics‐1
TNF‐α: tumor necrosis factor α
Ubo: Ubo‐QIC
BPTES: bis‐2‐(5‐phenylacetamido‐1,3,4‐thiadiazol‐2‐yl)ethylsulfide
IP_3_
: inositol‐1,4,5‐triphosphate
ERK: extracellular‐sensing regulated kinase 1/2
PIP_2_
: phosphatidylinositol‐4,5‐bisphosphate

## Introduction

Metabolites are intermediates of biochemical pathways, serve as energy substrates, and regulate enzymatic pathways, but may also act as specific agonists at G protein‐coupled receptors (GPCRs). Succinate (SUC) is a central metabolite, constantly generated in the mitochondrial tricarboxylic acid (TCA) cycle, which specifically activates the succinate receptor 1 (SUCNR1).

It is widely recognized that both intra‐ and extracellular SUC levels increase when oxygen supply and energy demand are imbalanced. Conditions of such metabolic stress include, for example, hypoxia or inflammation, as well as physical exercise [1, 2, 3, 4]. Intra‐ and extracellular SUC concentrations are assumed to be high enough to activate SUCNR1, depending on the cellular context and metabolic state [5]. SUC may also be derived from gut microbiota, further adding to the complexity of SUC as a metabolite [6].

SUCNR1 is abundantly expressed in immune cells [7] and different organs and tissues, including the kidney [8], liver [9], and heart [10]. The known roles of SUCNR1 include its involvement in regulating blood pressure via the renin‐angiotensin system [11] and its importance for macrophage function [12].

SUCNR1 is a G_i_‐ and G_q_‐coupled receptor, although several studies could not confirm G_q_‐coupling and report G_i_ activation as the primary signaling pathway [13, 14]. A cell‐type‐ and context‐dependent signal transduction of SUCNR1 might explain these controversial results [5].

Upon agonist stimulation, most GPCRs are rapidly internalized and desensitized, which may terminate signaling or, in some cases, induce persistent intracellular signaling [15, 16, 17]. Extracellular and cell membrane‐impermeable ligands induce signaling by binding to a receptor localized at the cell surface (reviewed in Refs [18, 19]). A more complex relationship between receptor location and signaling can be assumed for intracellularly produced ligands, such as SUC.

Spatially‐biased, also referred to as location‐dependent, signaling has been shown for various GPCRs, usually promiscuously coupling to more than one G protein family, that is, thyroid‐stimulating hormone receptor [15], parathyroid hormone receptor [16], and the sphingosine‐1‐phosphate receptor 1 (S1P1R) [17]. Unlike SUCNR1, these receptors commonly bind ligands that are not produced intracellularly by the same cell. Despite the confirmed role of SUCNR1 in human health and disease, limited information is available on the relationship between cellular metabolism and a potential spatially‐biased signaling of SUCNR1. In particular, the reported inconsistency in determining SUCNR1‐mediated G_q_‐coupling may be due to differences in its subcellular localization. Activation of G_q_ protein leads to phospholipase C β (PLCβ) activation and the hydrolysis of phosphatidylinositol‐4,5‐bisphosphate (PI_(4,5)_P_2_) to diacylglycerol (DAG) and inositol‐1,4,5‐trisphosphate (IP_3_). Although PLCβ isoforms are present in endosomes, PI_(4,5)_P_2_ concentrations are preferentially enriched at the plasma membrane, suggesting that receptor localization at the cell surface is necessary for G_q_‐mediated formation of IP_3_ and DAG [20].

We, therefore, hypothesized that changes in the cellular metabolic state cause alterations in both subcellular location and signaling of SUCNR1, which may explain why both pro‐ and anti‐inflammatory actions have been assigned to SUCNR1 signaling (reviewed in Refs [21, 22]). The mechanisms underlying these effects remain elusive, and pharmacological studies of SUCNR1 with non‐metabolite synthetic agonists are limited.

Previous work revealed a mutual dependence of SUCNR1 expression and cancer cell metabolism [23]. To address potential SUCNR1 signaling from endosomal compartments as opposed to signaling from the plasma membrane, we used HEK293‐T cells. We applied a broad set of bioluminescence resonance energy transfer (BRET)‐based biosensors in combination with dynamic mass redistribution (DMR) measurements, second messenger assays, and extracellular signal‐related kinase 1/2 (ERK) and Akt 1/2/3 phosphorylation analyses. THP‐1‐derived macrophages served as a model for endogenously SUCNR1‐expressing immune cells to analyze how the receptor's G_i_ and G_q_ protein signaling affect cellular energy homeostasis.

## Results

### SUCNR1 is intracellularly localized due to constant internalization and cellular SUC production

**Fig. 1:**
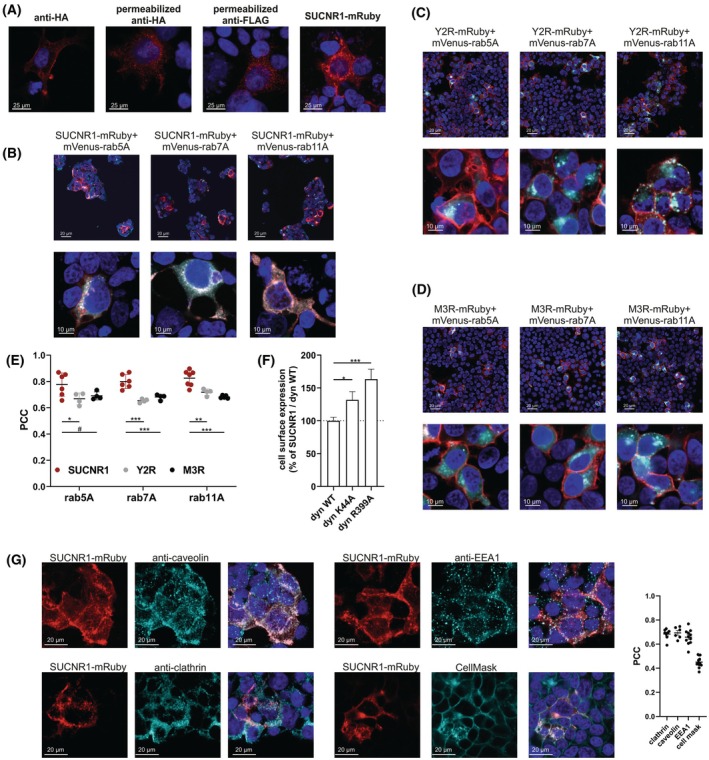
Subcellular distribution of SUCNR1 and dynamin‐2‐dependent internalization. (A) HEK293‐T cells were transfected with N‐terminally HA‐ and C‐terminally FLAG‐tagged SUCNR1 or C‐terminally mRuby‐tagged SUCNR1 (red). Cells were fixed and permeabilized as indicated. The N terminal HA‐tag was detected with a monoclonal anti‐HA antibody produced in mouse (1 : 1000), the C‐terminal FLAG‐tag with a monoclonal anti‐FLAG antibody produced in mouse (1 : 400), and a secondary Alexa Fluor555‐labeled (red) anti‐Mouse IgG antibody produced in goat (1 : 500). HEK293‐T cells were co‐transfected for confocal imaging with (B) SUCNR1‐mRuby, (C) Y2R‐mRuby, or (D) M3R‐mRuby (all red) and mVenus‐tagged marker proteins (cyan). (E) Co‐localization of each receptor with the respective subcellular marker was determined using the Pearson correlation coefficient (PCC) for *n* = 4 representative images (mean ± SEM). (F) Cell surface ELISA in HEK293‐T cells transiently co‐transfected with HA‐tagged SUCNR1 and with dynamin WT or internalization deficient mutants (dyn K44A, dyn R399A). Data are shown as % (mean ± SEM, *n* = 6) of HA‐tagged SUCNR1 co‐transfected with dynamin WT (OD = 0.096 ± 0.004). (G) Confocal images of transiently SUCNR1‐mRuby (red) transfected HEK293‐T cells were acquired. Cells were fixed, permeabilized and stained with primary anti‐caveolin (1 : 400), anti‐clathrin (1 : 1000), anti‐EEA1 antibody (1 : 100), and secondary antibodies (1 : 500) (anti‐clathrin: Alexa Fluor488 goat anti‐mouse; anti‐caveolin and anti‐EEA1: AlexaFluor488 goat anti‐rabbit). CellMask green plasma membrane stain was diluted 1 : 1000, and cells were imaged live. Co‐localization of SUCNR1‐mRuby with the respective subcellular marker was determined using the Pearson correlation coefficient (PCC) for *n* = 4 representative images (mean ± SEM). (A–E, G) Cell nuclei were stained with Hoechst 33342 (blue). Representative images of *n* = 3 experiments are shown, and 10 images were taken per experiment. (E, F) Statistical analyses were performed using an ordinary one‐way ANOVA. ^#^ *P* ≤ 0.1, *P ≤ 0.05, **P ≤ 0.01, ***P ≤ 0.001.

Using confocal microscopy, we found that SUCNR1 is localized to the plasma membrane and, to a great extent, to intracellular vesicles (Fig. 1A). This is in contrast to other receptors like the G_i_ protein‐coupled neuropeptide Y receptor 2 (Y2R) and the G_q_ protein‐coupled muscarinic receptor 3 (M3R), with neuropeptide Y (NPY) and the neurotransmitter acetylcholine as the endogenous agonist, respectively [24]. The preferred localization of Y2R and M3R is suitable for activation by extracellular ligands that are not constantly produced intracellularly, like SUC (Fig. 1B–D).

To dissect the subcellular localization of SUCNR1, we used confocal imaging and found significantly higher co‐localization of SUCNR1 with the endosomal marker proteins rab5A (early endosomes), rab7A (late endosomes), and rab11A (recycling endosomes) as compared to Y2R and M3R (Fig. 1B–E). ELISA analyses were used to determine whether the intracellular localization of SUCNR1 is due to internalization. The GTPase dynamin‐2 is involved in the membrane scission step during GPCR internalization [25]. Cells were either co‐transfected with SUCNR1 and dynamin‐2 wildtype (wt) or with SUCNR1 and the dynamin‐2 internalization‐deficient mutants K44A and R399A (Fig. 1F). In comparison to dynamin‐2 wt, we observed increased cell surface expression of SUCNR1 with K44A or R399A present, suggesting that SUCNR1 internalization is dynamin‐2‐dependent (Fig. 1F).

Further, subcellular localization analyses of SUCNR1 using monoclonal antibodies against marker proteins revealed the co‐localization of caveolin‐1, clathrin, and the early endosome antigen 1 (EEA‐1) with SUCNR1 to some extent (Fig. 1G). The CellMask Green Plasma Membrane Stain was also found intracellularly in cells expressing SUCNR1 (Fig. 1G). Together, these results suggest a constitutive SUCNR1 internalization, potentially due to the continuous production of SUC in cellular metabolism.

**Fig. 2:**
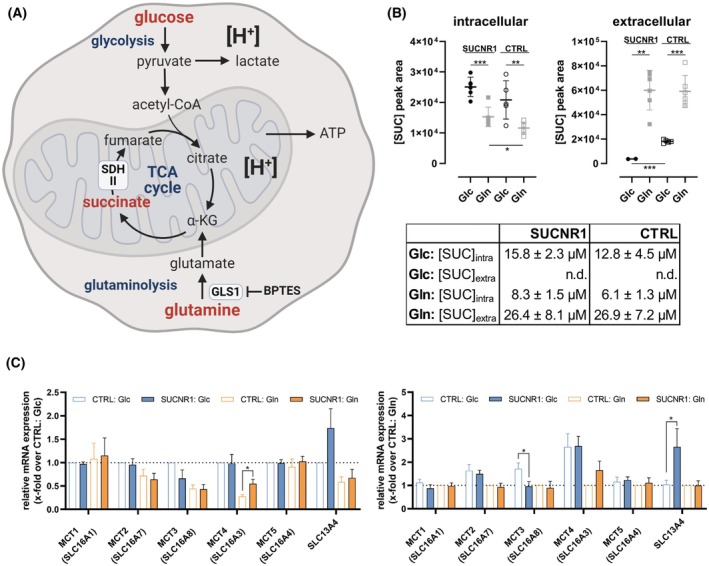
Succinate levels depend on the available energy substrate and SUCNR1. (A) Glucose (Glc) and glutamine (Gln) serve as energy substrates to generate succinate (SUC) in the TCA cycle. Gln is converted to glutamate by glutaminase (GLS1), followed by conversion to α‐ketoglutarate (α‐KG). Created with Biorender.com. (B) Gas chromatography–mass spectrometry analyses of SUCNR1‐expressing versus control (CTRL) HEK293‐T cells were performed subsequent incubation for 2 h in PBS with Glc or Gln. SUC concentrations are shown as peak area determined in cell lysates and medium (*n* = 6, shown as mean ± SD). Absolute SUC quantification was performed if SUC concentrations were sufficiently high and were indicated in μm. (C) RT‐qPCR experiments were performed to measure differences in the mRNA expression of potential SUC transporters in SUCNR1‐expressing versus CTRL HEK293‐T cells (*n* = 6, reference gene *ACTB C*_q_ = 16), shown as relative expression x‐fold over CTRL Glc or CTRL Gln as indicated (mean ± SEM). (A, C) Statistical analyses were performed using unpaired two‐tailed *t*‐tests. *P ≤ 0.05, **P ≤ 0.01, ***P ≤ 0.001.

Glucose (Glc) and glutamine (Gln) may both enter the TCA cycle to produce energy and SUC (Fig. 2A). Using gas chromatography–mass spectrometry (GC–MS), we analyzed how supplementation of medium with either 10 mm Glc or 2 mm Gln affects intra‐ and extracellular SUC levels in SUCNR1‐expressing HEK293‐T cells compared to cells without SUCNR1 expression (CTRL) (Fig. 2B). Relative SUC concentrations were determined as peak areas (Fig. 2B). Absolute quantification of SUC was performed when the signal strength was sufficient. In SUCNR1‐expressing and CTRL cells, these analyses revealed higher intracellular and lower extracellular SUC concentrations in Glc compared to Gln (Fig. 2B). This result implies that SUC is released from cells when Gln is present as an energy substrate, while SUC accumulates intracellularly with Glc as an energy substrate. Expression of SUCNR1 was associated with higher intracellular SUC levels in Gln and lower extracellular SUC levels in Glc (Fig. 2B).

We analyzed the mRNA expression levels of several potential SUC transporters of the monocarboxylate transporter (MCT, SLC16) and solute carrier 13 (SLC13) families (Fig. 2C). MCT2, MCT3, MCT4, and SLC13A4 mRNA expression is lower in Gln than in Glc, independent of receptor expression. Therefore, the reduced expression of SUC transporters might be responsible, at least to some extent, for the lower intra‐ and higher extracellular SUC concentrations in the Gln‐containing medium (Fig. 2B,C). In the Glc‐containing medium, SUCNR1‐expressing cells have higher SLC13A4 and lower MCT3 mRNA levels, which may explain their lower extracellular (and higher intracellular) SUC concentrations compared to CTRL cells (Fig. 2B,C). In Gln, MCT4 mRNA expression is higher in SUCNR1‐expressing cells, which may account for their higher intracellular SUC levels. These results imply that SUCNR1 is potentially involved in the transcriptional regulation of SUC‐transporting proteins, affecting intra‐ and extracellular SUC concentrations.

### SUCNR1 ligands differentially affect signal transduction and regulate cellular metabolic parameters

Besides SUC, *cis*‐epoxysuccinate (CES) and (6‐(4‐(Trifluoromethoxy)phenyl)picolinoyl)‐l‐aspartic acid (compound **31**) have been described as synthetic surrogate, non‐metabolite SUCNR1 agonists [26, 27].

We performed DMR analyses to evaluate the signaling properties of these non‐metabolite ligands compared to SUC and other metabolites. The responses recorded with the DMR technology reflect a sum of signaling events induced by the exogenous stimulation of a GPCR with its agonists [28]. Changes in intracellular mass near the plasma membrane are measured as a shift in wavelength of the reflected light in picometer (pm) over time, thus enabling conclusions about GPCR activation kinetics independent of the activated G protein.

**Fig. 3:**
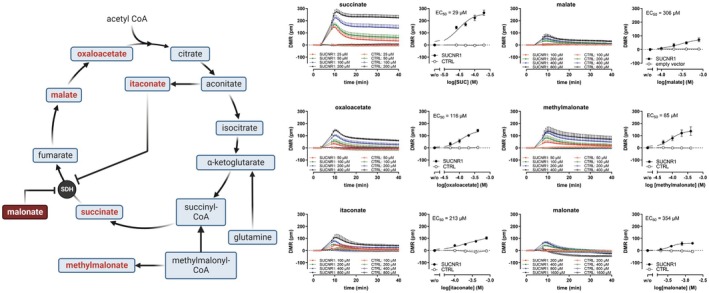
TCA cycle metabolites and succinate dehydrogenase (SDH) inhibitors activate SUCNR1. Left: The TCA cycle is a central mitochondrial metabolic pathway in most eukaryotic cells. Some cells are able to produce itaconate by converting aconitate. Both itaconate and malonate are inhibitors of SDH. Highlighted in red are metabolites that activate SUCNR1. Created with Biorender.com. Right: HEK293‐T cells were transiently transfected with SUCNR1. DMR measurements were performed upon stimulation with increasing concentrations of TCA cycle intermediates (succinate, malate, oxaloacetate, and methylmalonate) or SDH inhibitors (itaconate and malonate). Data are shown as mean ± SEM of *n* = 3 independent experiments, each carried out in triplicates.

**Fig. 4:**
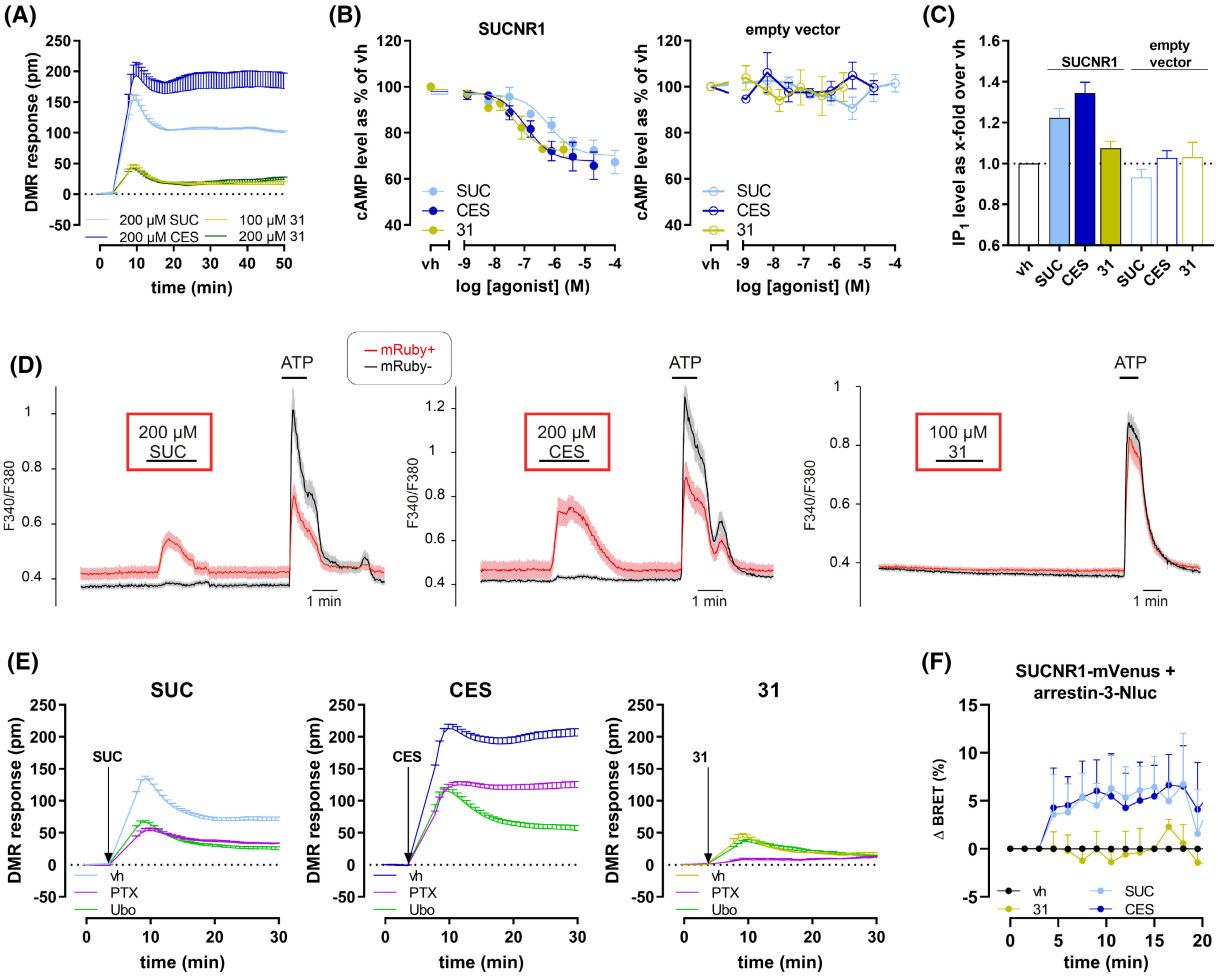
Characterization of the synthetic SUCNR1 ligand compound **31**. For DMR measurements (A, E each *n* = 3), cAMP assays (*n* = 3) (B), and IP_1_ assays (*n* = 4) (C), HEK293‐T were transfected with SUCNR1 and stimulated with 100 μm of succinate (SUC), *cis*‐epoxysuccinate (CES) or compound **31**. (D) HEK293‐T cells were transfected with SUCNR1‐mRuby. The fluorescence ratio (F340/F380) represents the time course of calcium responses in SUCNR1‐mRuby transfected (mRuby+) and non‐transfected (mRuby−) cells from the same coverslip. Each trace represents the average calcium signal ± SEM of SUC: 32 cells, CES: 28 cells, **31**: 37 cells. The application of ATP (100 μm) served as the stimulus for the endogenous calcium‐releasing pathway. (E) For DMR measurements, cells were incubated overnight with the G_i_ inhibitor pertussis toxin (PTX) or 30 min before the assay with the G_q_ inhibitor UBO‐QIC (Ubo). Cells were stimulated with 100 μm SUC, CES, or compound **31**. Untreated cells served as control (vehicle, vh). (F) For BRET analyses, HEK293‐T cells were transfected with SUCNR1‐mVenus and arrestin‐3‐Nluc (*n* = 4). BRET ratios were defined as acceptor emission/donor emission. (A–C, E, F) Data are shown as mean ± SEM of the indicated number of independent experiments, each carried out in triplicates.

Metabolite SUCNR1 agonists (malate, oxaloacetate, methylmalonate, itaconate, and malonate) showed concentration‐dependent responses upon SUCNR1 stimulation, although both potency and efficacy were lower than that determined for SUC (Fig. 3). That is in contrast to the low signal we observed upon application of compound **31** (Fig. 4A). However, cAMP inhibition assays revealed a potency (EC_50_ ~ 47 nm, 95% CI: 15.4–146 nm) and efficacy of compound **31** comparable to that of CES (EC_50_ ~ 111 nm, 95% CI: 29.6–417 nm), while the potency for SUC (EC_50_ ~ 644 nm, 95% CI: 170–2436 nm) was about 6‐fold lower than that of CES (Fig. 4B). When analyzing the G_q_ protein‐activated pathways, we found that SUC‐ and CES‐induced SUCNR1‐mediated IP_1_ formation and Ca^2+^ release, but this was not observed upon stimulation with compound **31** (Fig. 4C,D). This is supported by the fact that the compound **31**‐induced DMR signal was only sensitive to the G_i_ inhibitor pertussis toxin (PTX) [29] but not to the G_q_ inhibitor UBO‐QIC (FR900359, Ubo) [30, 31], while the SUC‐ and CES‐induced signals were sensitive to both PTX and Ubo (Fig. 4E). This experimental setup allows for untangling the contribution of G_i_ and G_q_ signal transduction pathways to the SUCNR1 overall signaling.

SUCNR1 has been shown to only weakly recruit arrestins [26, 32]. Using BRET, we measured arrestin‐3 recruitment upon SUCNR1 stimulation with SUC, CES, and compound **31** (Fig. 4F). While both SUC and CES caused an increase in ΔBRET, no arrestin‐3 interaction was observed upon stimulation with compound **31** (Fig. 4F).

In conclusion, compound **31** induces SUCNR1‐mediated cAMP inhibition with high potency, while 100 μm **31** failed to activate G_q_ protein or induce arrestin‐3 recruitment in our experimental setups (Fig. 4). Consequently, we used CES as a non‐metabolite agonist in comparison to SUC for our further analyses.

**Fig. 5:**
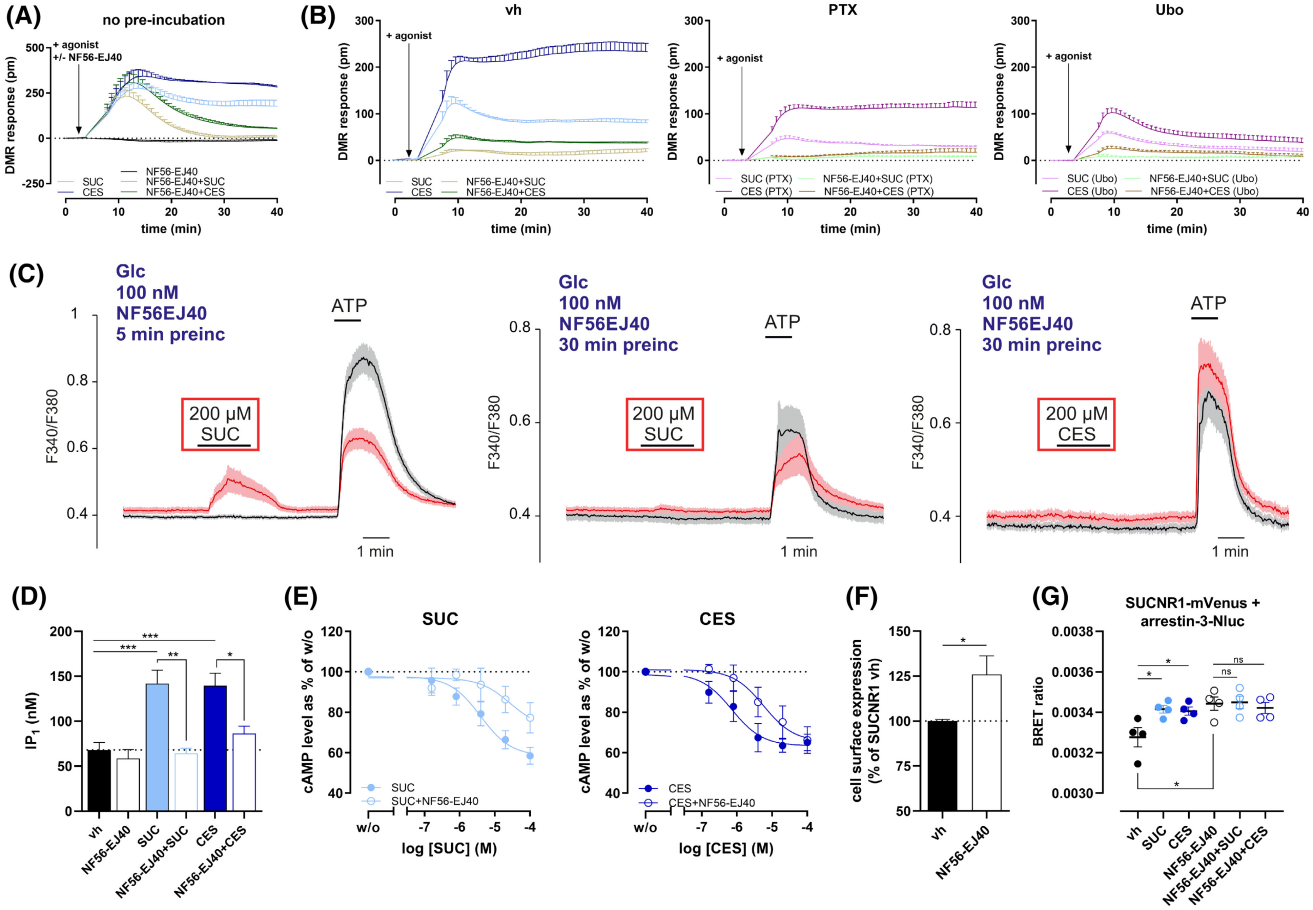
Evaluation of the SUCNR1 antagonist NF56‐EJ40. For DMR measurements (A, B), IP_1_ assays (D), cAMP assays (E), and cell surface ELISA (F), HEK293‐T were transfected with SUCNR1. (A) DMR measurements were performed, simultaneously applying *cis*‐epoxysuccinate (CES) or succinate (SUC) (each 100 μm) and 100 nm of NF56‐EJ40 (*n* = 4). (B) Cells were pre‐incubated for 30 min with or without the SUCNR1 antagonist 100 nm NF56‐EJ40. Cells were incubated for 16 h before the assay with or without the G_i_ protein inhibitor pertussis toxin (PTX, 100 ng·mL^−1^, middle graph). Cells were pre‐incubated for 30 min with or without the G_q_ protein inhibitor UBO‐QIC (Ubo, 300 nm, right graph) (*n* = 4). (C) HEK293‐T cells were transfected with SUCNR1‐mRuby. Cells were pre‐incubated for the indicated time with the SUCNR1 antagonist (100 nm NF56‐EJ40) and stimulated with SUC and CES, as shown. The application of ATP (100 μm) served as the stimulus for the endogenous calcium‐releasing pathway. Each trace represents the average calcium signal ± SEM of left: 24 cells, middle: 33 cells, right: 35 cells. (D, E) Cells were pre‐incubated for 30 min with or without (vh) 100 nm NF56 EJ40. (D) As indicated, IP_1_ accumulation (*n* = 4) and (E) cAMP inhibition assays (*n* = 5) were performed by stimulating with SUC or CES. (F) Cell surface ELISA was performed in cells pre‐incubated for 30 min with 100 nm NF56‐EJ40 or without (vh) (*n* = 3). (G) HEK293‐T cells were transiently co‐transfected with SUCNR1‐mVenus and arrestin‐3‐Nluc. Cells were 30 min pre‐incubated with 100 nm NF56‐EJ40 or without (vh) and stimulated with 200 μm SUC or CES (*n* = 4). BRET ratios were defined as acceptor emission/donor emission. (D, F, G) Statistical analyses were performed using a one‐way ANOVA and unpaired two‐tailed *t*‐tests, respectively. (A, B, D–G) All data are shown as mean ± SEM of the indicated number of independent experiments, each carried out in triplicates. *P ≤ 0.05, **P ≤ 0.01, ***P ≤ 0.001.

Haffke *et al*. [33] identified 2‐(2‐(4′‐((4‐methylpiperazin‐1‐yl)methyl)‐[1,1′‐biphenyl]‐3‐carboxamido)phenyl)acetic acid (NF56‐EJ40) as a human SUCNR1‐selective antagonist, which we also evaluated using DMR analyses. NF56‐EJ40 alone did not induce a significant DMR signal (Fig. 5A). Simultaneous addition of the SUCNR1 antagonist NF56‐EJ40 (100 nm) and SUCNR1 agonists SUC and CES caused a reduction of the DMR signal after about 20 min (Fig. 5A). If cells were pre‐incubated for 30 min with 100 nm NF56‐EJ40, a DMR response was still detectable upon stimulation with SUC or CES, although it was about 5‐fold lower than in the absence of the antagonist (Fig. 5B). When the cells were pre‐incubated simultaneously with 100 nm NF56‐EJ40 and the G_i_ inhibitor PTX, no agonist‐induced DMR signal was observed. A residual SUC‐ and CES‐mediated DMR signal was still observable following pre‐incubation with the G_q_ inhibitor Ubo and 100 nm NF56‐EJ40 (Fig. 5B). However, no SUCNR1‐dependent calcium response could be detected upon the addition of agonists if cells were pre‐incubated for 30 min with NF56‐EJ40 (Fig. 5C). NF56‐EJ40 also completely inhibited the agonist‐induced SUCNR1‐mediated IP_1_ formation and caused a significant shift of the cAMP inhibition curve (Fig. 5D,E). All subsequent experiments involving NF56‐EJ40 were performed after 30 min pre‐incubation of the cells.

SUC is constantly produced in cells (Fig. 2A), and the observed intracellular (co)localization of SUCNR1 with endosomal marker proteins and plasma membrane stain suggests its constant internalization (Fig. 1). We used ELISA analyses and found an increased SUCNR1 plasma membrane expression when cells were pre‐incubated with NF56‐EJ40 (Fig. 5F). SUC and CES increased the BRET ratio between SUCNR1‐mVenus and arrestin‐3‐Nluc, as did NF56‐EJ40 (Fig. 5G). Thus, NF56‐EJ40 inhibits SUCNR1 internalization and increases SUCNR1 interaction with arrestin‐3 at the plasma membrane. No additional increase in the BRET ratio is observed with SUC and CES in the presence of NF56‐EJ40. In summary, the antagonist inhibits SUCNR1 signaling, and compound **31** potently activates SUCNR1‐mediated G_i_ signaling.

### Reduced G_q_ but not G_i_ signaling of SUCNR1 in the presence of Gln

**Fig. 6:**
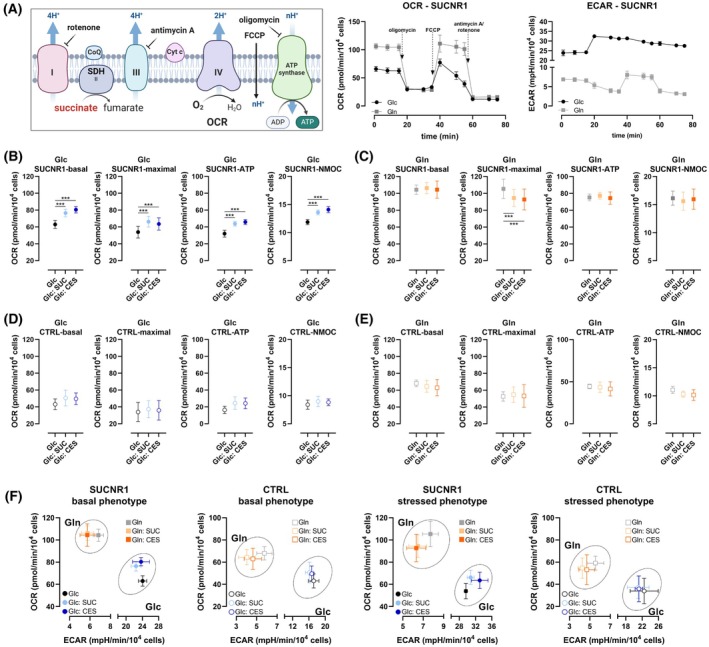
SUCNR1 agonists increase respiratory parameters in SUCNR1‐expressing HEK293 cells in Glucose (Glc). (A) The electron transport chain (ETC) is located in the inner mitochondrial membrane, consisting of several protein complexes, ultimately leading to the production of ATP. Complexes I, III, and IV translocate protons (H^+^). Complex II is succinate dehydrogenase (SDH). Schematic created using Biorender.com. Oxygen consumption rate (OCR) and extracellular acidification rate (ECAR) were measured with the Seahorse XFe96 extracellular flux analyzer in the presence of 10 mm Glc or 2 mm glutamine (Gln) and different specific inhibitors of the ETC as depicted. Changes in OCR and ECAR over time are depicted for SUCNR1‐expressing cells. (B–F) Cells were stimulated with 200 μm succinate (SUC) or *cis*‐epoxysuccinate (CES). (B) In Glc, stimulation of SUCNR1‐expressing cells with agonists results in increased respirational parameters, as indicated. (C) In Gln, stimulation with SUC or CES caused a decrease in maximal respiration in SUCNR1‐expressing cells. Stimulation with SUC or CES did not affect basal and maximal respiration of HEK293‐CTRL cells in (D) Glc or (E) Gln. (F) In Glc, stimulation with SUC or CES induced a detectable shift in basal and stressed metabolic phenotype in SUCNR1‐expressing cells, which was absent in CTRL cells. In Gln, a shift in the opposite direction was found in the stressed metabolic phenotype in SUCNR1‐expressing cells. Data are shown as mean ± SEM of *n* = 3 independent experiments, each carried out in 6 technical replicates. (B, C) Statistical analyses were performed by applying two‐way repeated measures ANOVAs. ***P ≤ 0.001.

We used CES as a synthetic agonist besides SUC to further investigate the effects of SUCNR1 activation on cellular metabolism. The Seahorse XF Cell Mito Stress Test was carried out in the presence of Glc or Gln as energy substrates. This assay is based on the sequential addition of specific electron transport chain inhibitors (Fig. 6A). Upon SUC or CES stimulation in the presence of Glc, we found an increase in basal and maximal oxygen consumption rate (OCR, basal and stressed phenotype) as well as ATP production and non‐mitochondrial oxygen consumption (NMOC) in SUCNR1‐expressing but not in control (CTRL) cells (Fig. 6B–D). In the Gln‐containing medium, all respirational parameters were higher for the SUCNR1 and the CTRL cells. SUCNR1 stimulation with SUC and CES decreased maximal respiration only in the SUCNR1‐expressing cell line, an effect not observed in the CTRL cells (Fig. 6C,E). Plotting the extracellular acidification rate (ECAR) and the OCR under basal and stressed conditions revealed changes in the metabolic phenotype. In the Glc‐containing medium, a shift toward a more aerobic phenotype of stimulated SUCNR1‐expressing cells was observed under basal conditions. In contrast, in the Gln‐containing medium, a shift toward a more quiescent phenotype was found under stressed conditions (Fig. 6F). These results suggest that the energy substrate‐dependent signaling of SUCNR1 impacts cellular energy homeostasis.

**Fig. 7:**
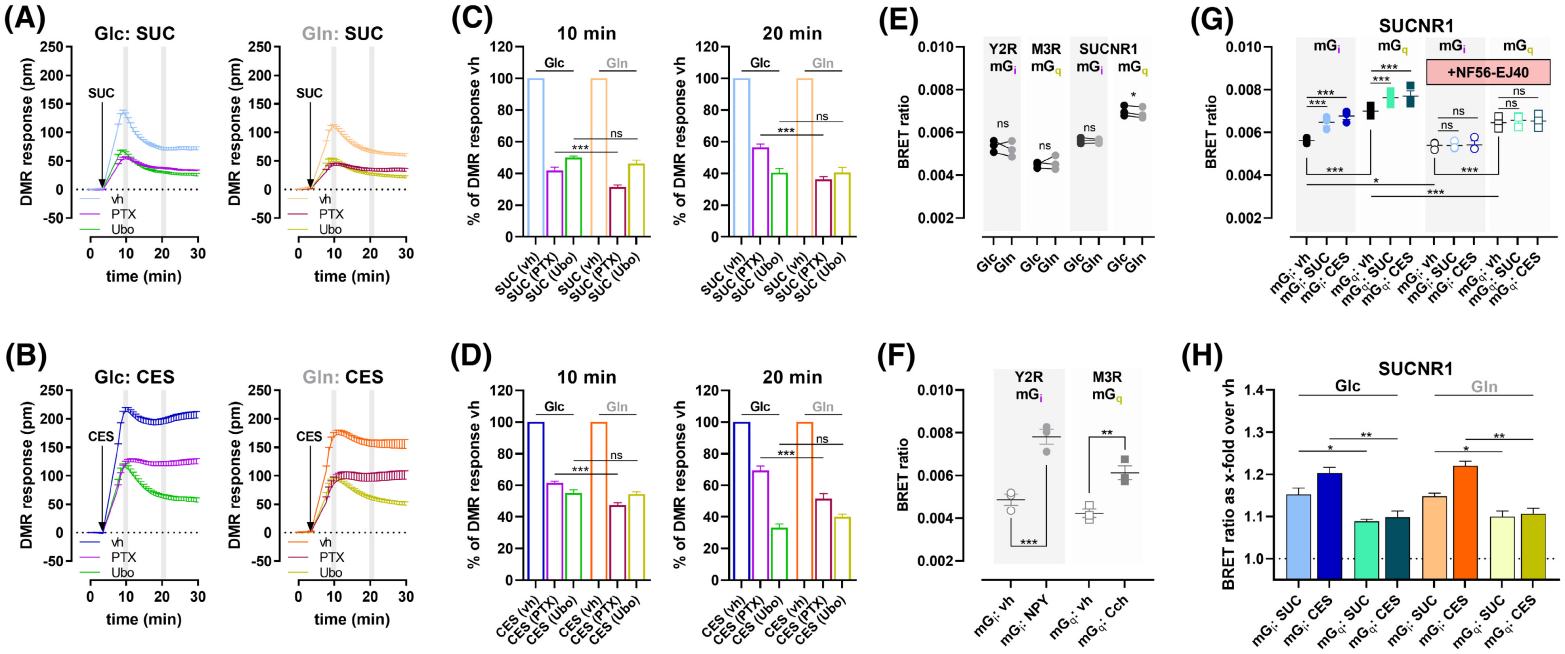
SUCNR1‐mediated signaling is affected by the availability of different energy substrates, causing differential effects on cellular metabolism. (A–D) Dynamic mass redistribution (DMR) measurements in SUCNR1‐transfected HEK293‐T cells in the presence of Glc or Gln, stimulated with 100 μm succinate (SUC) or *cis*‐epoxysuccinate (CES). Cells were either untreated (vh), incubated for 16 h before the assay with the G_i_ protein inhibitor pertussis toxin (PTX, 100 ng·mL^−1^), or for 30 min before the assay with 300 nm of the G_q_ protein inhibitor UBO‐QIC (Ubo). Data are shown as mean ± SEM of *n* = 4 independent experiments. (A, C) Stimulation with SUC in Glc and Gln. (B, D) Stimulation with CES in Glc and Gln. (C, D) Data shown in the absence of inhibitor (vh) was set to 100% in each medium. (E–H) As indicated, BRET assays were performed in cells co‐transfected with Y2R‐Nluc, M3R‐Nluc, or SUCNR1‐Nluc, and mVenus‐tagged miniG_i_ or miniG_q_ protein. (E) Cells were incubated for 30 min in PBS Glc or PBS Gln. (F) Y2R was stimulated with 1 μm neuropeptide Y (NPY) and M3R with 100 μm carbachol (Cch), each serving as a control for G_i_ and G_q_ signaling, respectively. (G) SUCNR1 was stimulated with 200 μm SUC or CES with or without preincubation with 100 nm of the SUCNR1 antagonist NF56‐EJ40. (E–G) BRET ratios before and after agonist stimulation are shown as mean ± SEM of *n* = 3 independent experiments, each carried out in triplicates. Statistical analyses were performed using repeated measures of one‐way ANOVA or paired two‐tailed *t*‐tests, respectively. (H) BRET ratios for SUC and CES are shown as x‐fold over vehicle (vh) control in Glc and Gln (mean ± SEM, *n* = 3). Statistical analyses were performed using unpaired two‐tailed *t*‐tests. BRET ratios were defined as acceptor emission/donor emission. ns, not significant; *P ≤ 0.05, **P ≤ 0.01, ***P ≤ 0.001.

We used DMR measurements to investigate the relative contribution of G_i_‐ and G_q_‐dependent signaling in Glc‐ versus Gln‐containing medium by application of PTX or Ubo, respectively. PTX and Ubo caused a significant reduction (but not a complete inhibition) of the SUC or CES‐induced SUCNR1‐dependent DMR response in both Glc and Gln (Fig. 7A,B). We set the respective agonist‐induced signal (at ~ 10 and ~ 20 min) in the absence of inhibitor (vehicle, vh) to 100% to show the relative contribution of G_i_ and G_q_ to SUCNR1‐induced signaling in the respective medium (Fig. 7C,D). When the cells were incubated with PTX, which inhibits the G_i_‐mediated SUCNR1 response, we observed that the remaining DMR response was significantly decreased in Gln compared to Glc (Fig. 7C,D). This, in turn, indicates a lower G_q_‐mediated response of SUCNR1 in the presence of Gln. In contrast, the G_i_‐mediated response of SUCNR1 to its agonists was unaffected by the energy substrate, as the relative signal did not differ in the presence of Ubo (Fig. 7C,D). These results imply diminished G_q_‐mediated SUCNR1 signaling in Gln.

To further validate those findings, we used miniG proteins, which are stable (and engineered) cytosolic GTPase domains of Gα subunits that lack the Gβγ‐binding surface [34, 35]. MiniG proteins functionally mimic the GPCR‐bound state of G protein heterotrimers and, in unstimulated cells, may partially reflect the binding of miniG proteins to a ligand‐free receptor [34, 35]. Y2R and M3R were included as controls for G_i_‐ and G_q_‐coupling, respectively, to exclude non‐specific effects of Glc or Gln on miniG protein recruitment. We found that cytosolic miniG_q_ protein recruitment was significantly reduced in Gln as compared to Glc for SUCNR1, while effects on miniG protein recruitment dependent on energy substrate were absent for Y2R and M3R (Fig. 7E). Y2R stimulated with NPY increased BRET ratio with cytosolic miniG_i_, and stimulation of M3R with Cch caused a BRET ratio increase with cytosolic miniG_q_ (Fig. 7F). Similarly, SUCNR1 stimulation with SUC and CES caused an increase in BRET ratio with both cytosolic miniG_i_ and miniG_q_ (Fig. 7G). The basal BRET ratio of SUCNR1 was significantly higher with miniG_q_ than miniG_i_ (Fig. 7G). Pre‐incubation with the antagonist NF56‐EJ40 caused a reduction of the BRET ratio in the absence of an agonist, while the addition of SUC or CES could not increase the BRET ratio anymore (Fig. 7G). The relative change in the BRET ratio inducible by SUC or CES was significantly higher for SUCNR1 with miniG_i_ than with miniG_q_ (Fig. 7H). Thus, we conclude that SUCNR1 shows stronger coupling to G_q_ proteins under basal conditions, while upon stimulation, its coupling to G_i_ proteins is stronger (Fig. 7G,H).

**Fig. 8:**
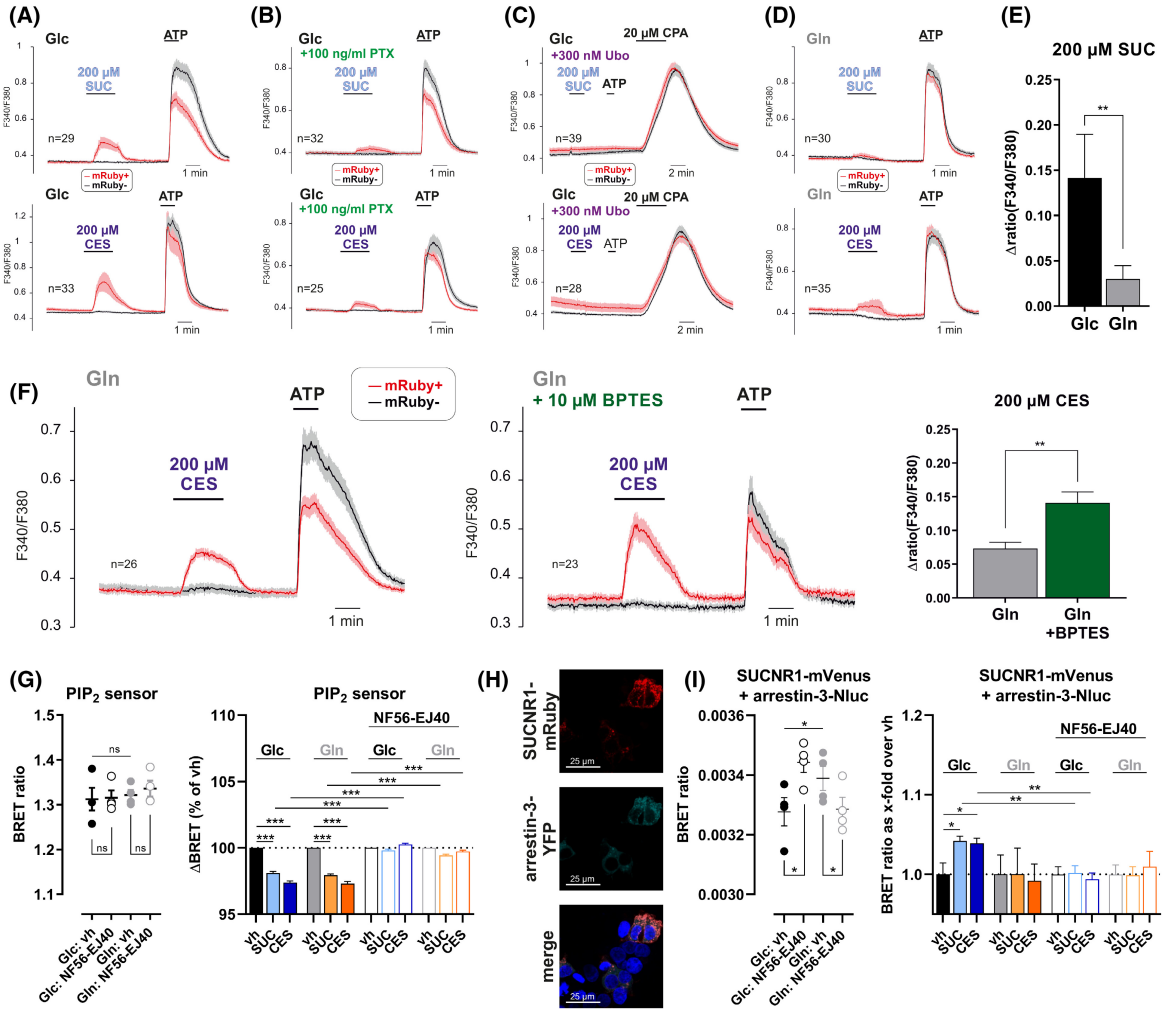
SUCNR1‐mediated G_i_ and G_q_ signaling depends on the available energy substrates and localization. (A–F) The fluorescence ratio (F340/F380) represents the time course of calcium responses in SUCNR1‐mRuby transfected (mRuby+) and non‐transfected (mRuby−) cells from the same coverslip. Each trace represents the average calcium signal ± SEM of the indicated number of cells. Application of ATP (100 μm) or the sarcoendoplasmic reticulum calcium ATPase blocker cyclopiazonic acid (CPA, 20 μm) served as stimuli for endogenous calcium‐releasing pathways. As indicated, cells were stimulated with succinate (SUC) and *cis*‐epoxysuccinate (CES) in (A) Glc and, in addition, either (B) incubated for 16 h before the measurement with the G_i_ protein inhibitor pertussis toxin (PTX, 100 ng·mL^−1^), or (C) for 30 min before the assay with 300 nm of the G_q_ protein inhibitor UBO‐QIC (Ubo). (D) Cells were stimulated with SUC or CES in Gln. (E) Calcium signals were quantified using the Δratio(F340/F380) from basal and peak amplitude before and after agonist application (mean ± SD, *n* = 4) for data shown in (A) and (D). (F) Following incubation in a medium supplemented with 2 mm Gln with or without the glutaminase inhibitor BPTES, cells were stimulated with CES as indicated. Calcium signals were quantified using the Δratio(F340/F380) from basal and peak amplitude before and after agonist application (mean ± SD, *n* = 3). HEK293‐T were transiently transfected with (G) SUCNR1 and the PIP_2_ sensor, (H) SUCNR1‐mRuby and arrestin‐3‐YFP, or (I) SUCNR1‐mVenus and arrestin‐3‐Nluc. Assays were performed in PBS with glucose (Glc) or glutamine (Gln). Cells were stimulated with 200 μm SUC or CES as indicated. (G, I) BRET ratios were defined as acceptor emission/donor emission. Data are shown as mean ± SEM of *n* ≥ 3 independent experiments. (G, I) Statistical analyses with BRET data were performed using paired two‐tailed *t*‐tests. Statistical analyses of Calcium data were performed using unpaired two‐tailed *t*‐tests (E, F). Baseline‐corrected BRET data (G, I) was analyzed using a one‐way ANOVA. *P ≤ 0.05, **P ≤ 0.01, ***P ≤ 0.001.

We measured calcium responses and used a biosensor for PI_(4,5)_P_2_ to investigate downstream effectors of the G_q_ pathway [36]. Ca^2+^ was released upon stimulation with SUC and CES in Glc, an effect strongly inhibited by the G_i_ inhibitor PTX and completely abolished by the G_q_ inhibitor Ubo (Fig. 8A–C). Hence, Ca^2+^ signaling of SUCNR1 also requires activation of G_i_, although G_q_ proteins ultimately control it. In the Gln‐supplemented medium, SUCNR1‐mediated Ca^2+^ signals were significantly lower than those obtained in Glc (Fig. 8D,E). We used Bis‐2‐(5‐phenylacetamido‐1,2,4‐thiadiazol‐2‐yl)ethyl sulfide (BPTES) [37], which is an allosteric inhibitor of glutaminase (GLS1) to link Gln‐dependent metabolism to diminished SUCNR1‐mediated G_q_ signaling. Conversion of Gln to glutamate by GLS1 is followed by conversion to α‐ketoglutarate, which is then oxidized in the TCA cycle, a process termed glutaminolysis (Fig. 2A). Ca^2+^ responses in cells incubated in a Gln‐supplemented medium with or without BPTES revealed that inhibition of glutaminolysis results in increased SUCNR1‐mediated G_q_ signaling (Fig. 8F).

Using the PIP_2_ sensor, we found no significant differences for unstimulated SUCNR1 when comparing BRET ratios obtained in Glc or Gln (Fig. 8G). Agonist‐stimulation of SUCNR1 induced a comparable decrease in ΔBRET in Glc and Gln, which was not observed when cells were pre‐incubated with NF56‐EJ40 (Fig. 5G). SUCNR1‐mRuby co‐localizes to some extent with arrestin‐3‐YFP (Fig. 8H). Gln alone caused an increase in the BRET ratio between SUCNR1‐mVenus and arrestin‐3‐Nluc, which was inhibited by NF56‐EJ40 (Fig. 8I). In Gln, agonist stimulation could not induce a further increase in arrestin‐3 recruitment (Fig. 8I).

**Fig. 9:**
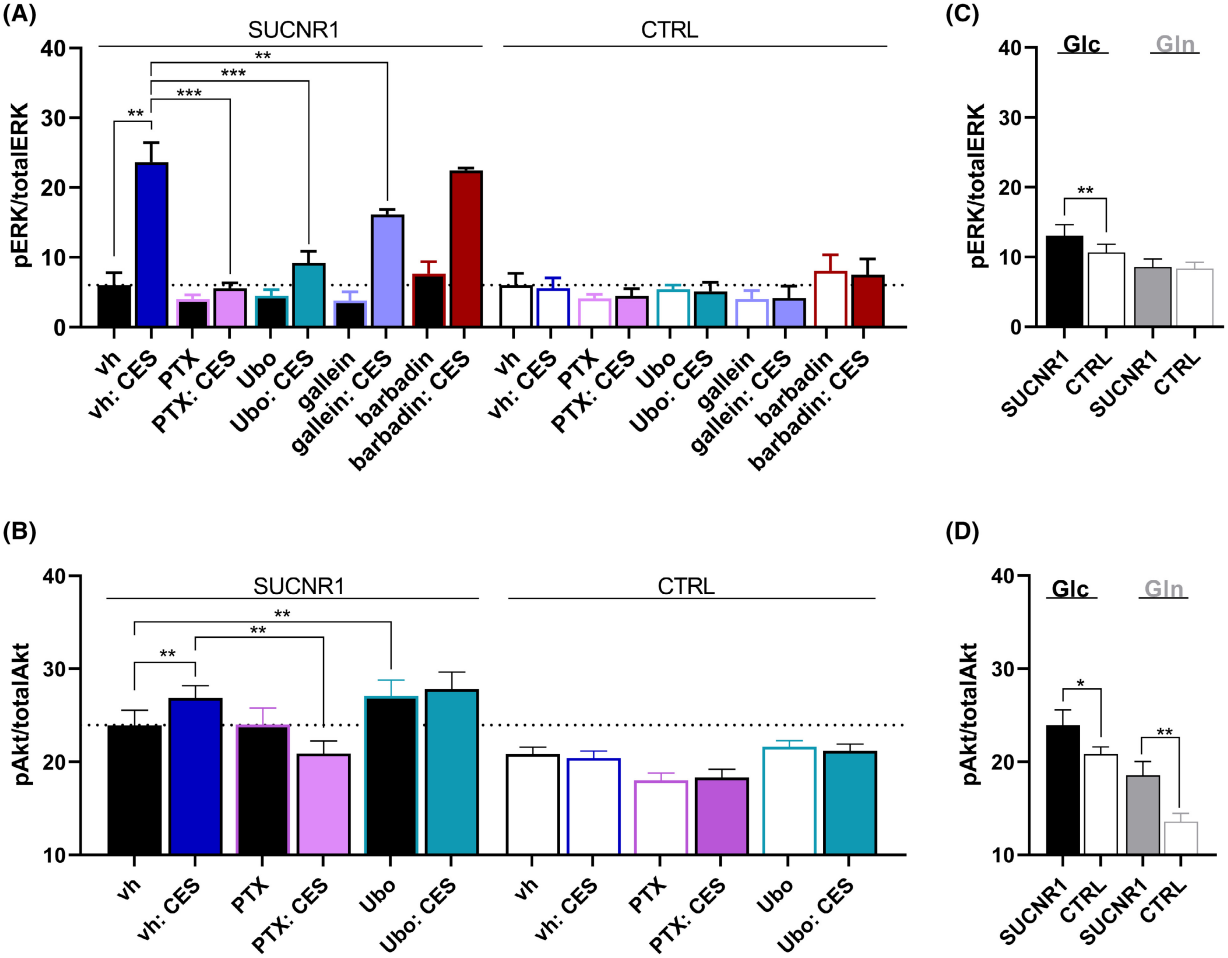
SUCNR1‐induced ERK and Akt signaling. Phosphorylated and total levels of ERK (A, C) and Akt1/2/3 (B, D) were measured upon stimulation with 200 μm *cis*‐epoxysuccinate (CES) in HEK293‐T cells transfected with SUCNR1 or empty vector (CTRL). (A, B) Assays were performed in the absence (vh) and the presence of inhibitors, incubated 16 h prior to the assay with 100 ng·mL^−1^ of the G_i_ protein inhibitor pertussis toxin (PTX), or 30 min before the assay with 300 nm of the G_q_ protein inhibitor UBO‐QIC (Ubo), or 50 μm of the Gβγ inhibitor gallein, or 100 μm barbadin as indicated (*n* = 3). Barbadin blocks agonist‐promoted arrestin‐dependent and clathrin‐mediated endocytosis. Phosphorylated and total ERK (C) and Akt1/2/3 (D) were measured after cells were incubated for 30 min in PBS with Glc or Gln (*n* = 5). Statistical analyses were performed using repeated measures of one‐way ANOVA (A, B) or paired two‐tailed *t*‐tests (C, D), respectively. Data are shown as mean ± SEM of the indicated number of independent experiments, each carried out in triplicates. *P ≤ 0.05, **P ≤ 0.01, ***P ≤ 0.001.

Phosphorylation of ERK may be induced by Gα_i_, Gα_q_ but also by Gβγ or arrestin‐3. We tested the contribution of each of those signaling components to agonist‐induced SUCNR1‐mediated ERK signaling by application of PTX, Ubo, gallein [38], and barbadin [39], as selective Gα_i_, Gα_q_, Gβγ, and arrestin/β2‐adaptin inhibitor, respectively. CES‐induced ERK1/2 phosphorylation was abolished by PTX, partially reduced by Ubo and gallein, and unaffected by barbadin (Fig. 9A). Thus, SUCNR1‐mediated ERK signaling strongly depends on Gα_i_ and to some extent on Gα_q_ and Gβγ, whereas arrestin‐3 is not involved. Similarly, we analyzed SUCNR1‐induced Akt‐phosphorylation. Stimulation with CES increased pAkt/total Akt SUCNR1‐dependently (Fig. 9B). This signal was unaffected by Ubo and completely diminished by PTX. However, in the absence of CES, PTX had no effect, and Ubo caused an increase in Akt phosphorylation, which was SUCNR1‐specific since no such effect was observed in CTRL cells. ERK and Akt signaling regulate metabolism (reviewed in Refs [40, 41]). We found that SUCNR1 expression is accompanied by increased Akt levels in Glc and Gln, and increased ERK levels in Glc (Fig. 9C,D).

From these results, we hypothesized that the intracellular localization of SUCNR1 is also affected by the available energy substrate.

We used BRET‐based analyses with SUCNR1‐Nluc and mVenus‐tagged marker proteins to determine if and how agonist stimulation affects SUCNR1 localization. Y2R and M3R, activated by non‐metabolite ligands, served as control receptors favoring G_i_‐ and G_q_‐coupling, respectively.

**Fig. 10:**
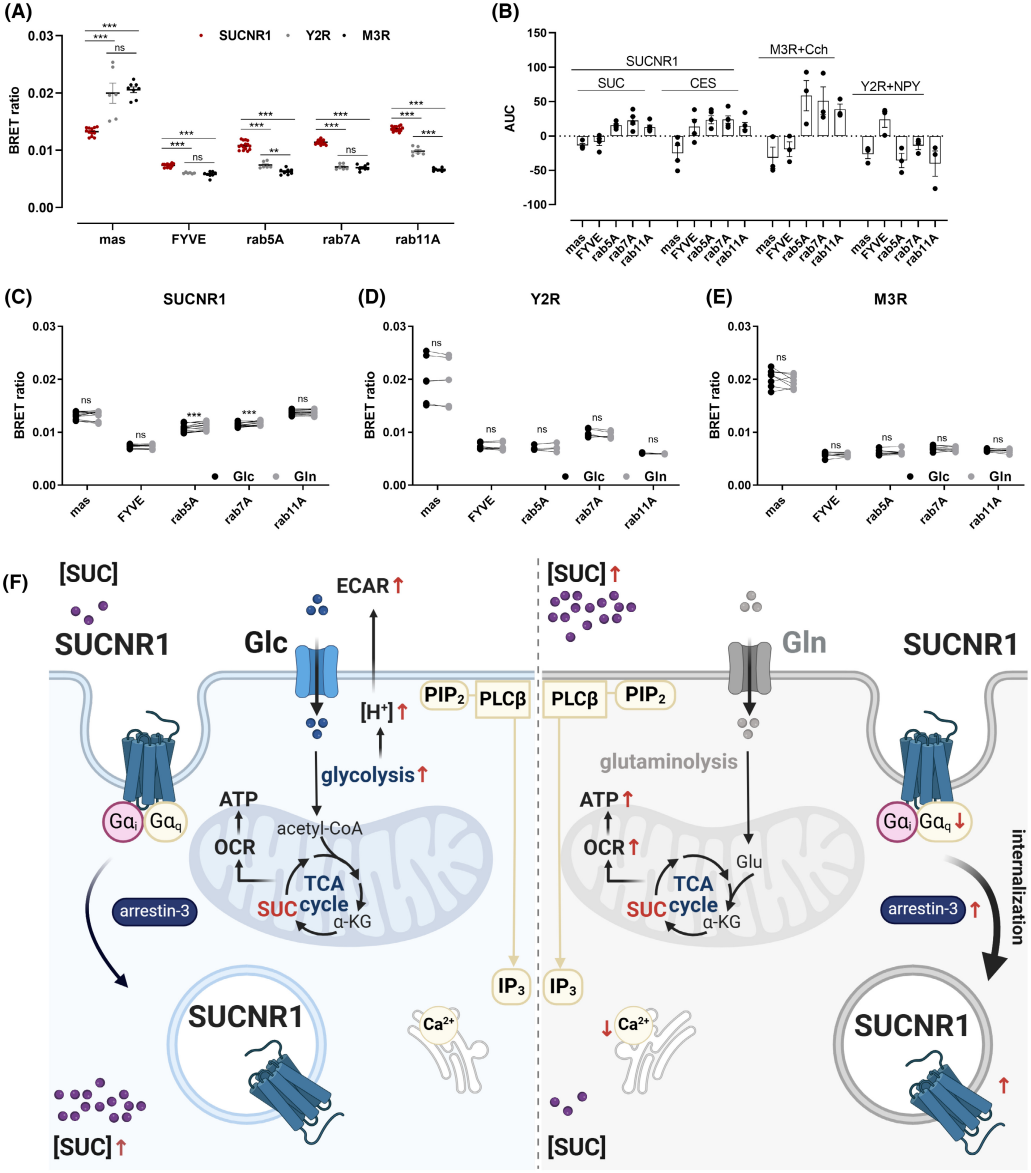
Changes in subcellular distribution of SUCNR1. For BRET analyses, HEK293‐T cells were transiently co‐transfected with SUCNR1‐Nluc, M3R‐Nluc, or Y2R‐Nluc and mVenus‐tagged subcellular marker proteins as indicated. BRET analyses were performed in PBS with glucose. (A) BRET ratios in the absence of agonists in Glc are shown (SUCNR1 mas, FYVE, each *n* = 12, rab5A, rab7A, rab11A, each *n* = 15, Y2R and M3R each *n* = 6). Statistical analyses were performed using an ordinary one‐way ANOVA. (B) Cells were stimulated with 200 μm succinate (SUC), 200 μm *cis*‐epoxysuccinate (CES), 100 μm carbachol (Cch), or 1 μm neuropeptide Y (NPY) as depicted. BRET ratios were defined as acceptor emission/donor emission, and the area under the curve (AUC) was extrapolated (SUCNR1 mas, FYVE, each *n* = 4, rab5A, rab7A, rab11A, each *n* = 5, Y2R and M3R each *n* = 3). BRET ratios in the absence of agonists comparing Glc versus Gln are shown for (C) SUCNR1 (mas, FYVE, each *n* = 12, rab5A, rab7A, rab11A, each *n* = 15), (D) Y2R (*n* = 6), and (E) M3R (*n* = 8). Statistical analyses were performed using paired two‐tailed *t*‐tests. ns, not significant; **P ≤ 0.01, ***P ≤ 0.001. Data are shown as mean ± SEM. (F) Graphical summary of findings on SUCNR1 signal transduction and influence on cellular metabolism depending on the available energy substrate. Created with Biorender.com.

In the absence of agonists, we found that BRET ratios with all endosomal marker proteins (FYVE, rab5A, rab7A, and rab11A) were significantly higher for SUCNR1 than for Y2R and M3R, while they were lower for SUCNR1 regarding plasma membrane localization (mas, Fig. 10A). Stimulation of SUCNR1‐Nluc and M3R‐Nluc with the respective agonists caused a decreased ∆BRET with membrane anchoring sequence (mas)‐mVenus and an increased ∆BRET with mVenus‐rab5A, ‐rab7A, and ‐rab11A (Fig. 10B). These results indicate increased internalization of both receptors upon agonist stimulation. A fraction of the receptors may undergo recycling, while another fraction may be routed toward degradation. Y2R stimulation with NPY caused a decrease in ∆BRET with mas, rab5A, rab7A, and rab11A proteins, and increased ∆BRET with FYVE (Fig. 10B). When comparing BRET ratios for SUCNR1 in Glc versus Gln, we observe increased values in Gln for rab5A and rab7A, an effect that is absent in Y2R‐ and M3R‐transfected cells (Fig. 10C–E). Thus, the presence of Gln as the energy substrate increases the presence of SUCNR1 in early and late endosomes, likely due to increased internalization.

In summary, we showed that the presence of Gln as the energy substrate is associated with a higher OCR and ATP production, and leads to increased extracellular SUC concentrations. SUCNR1‐mediated recruitment of cytosolic miniG_q_ protein is reduced in Gln while interaction with arrestin‐3 and residence in rab5A and rab7A endosomes are increased. Agonist‐induced SUCNR1‐mediated Ca^2+^ signals are reduced in Gln (Fig. 10F).

### SUCNR1 stimulation induces differential subcellular G_i_ and G_q_ signaling pattern

Since SUCNR1 is localized at the plasma membrane and, to a great extent, in endosomes, we analyzed whether SUCNR1 signaling differs depending on its respective localization. For this purpose, we chose several approaches.

**Fig. 11:**
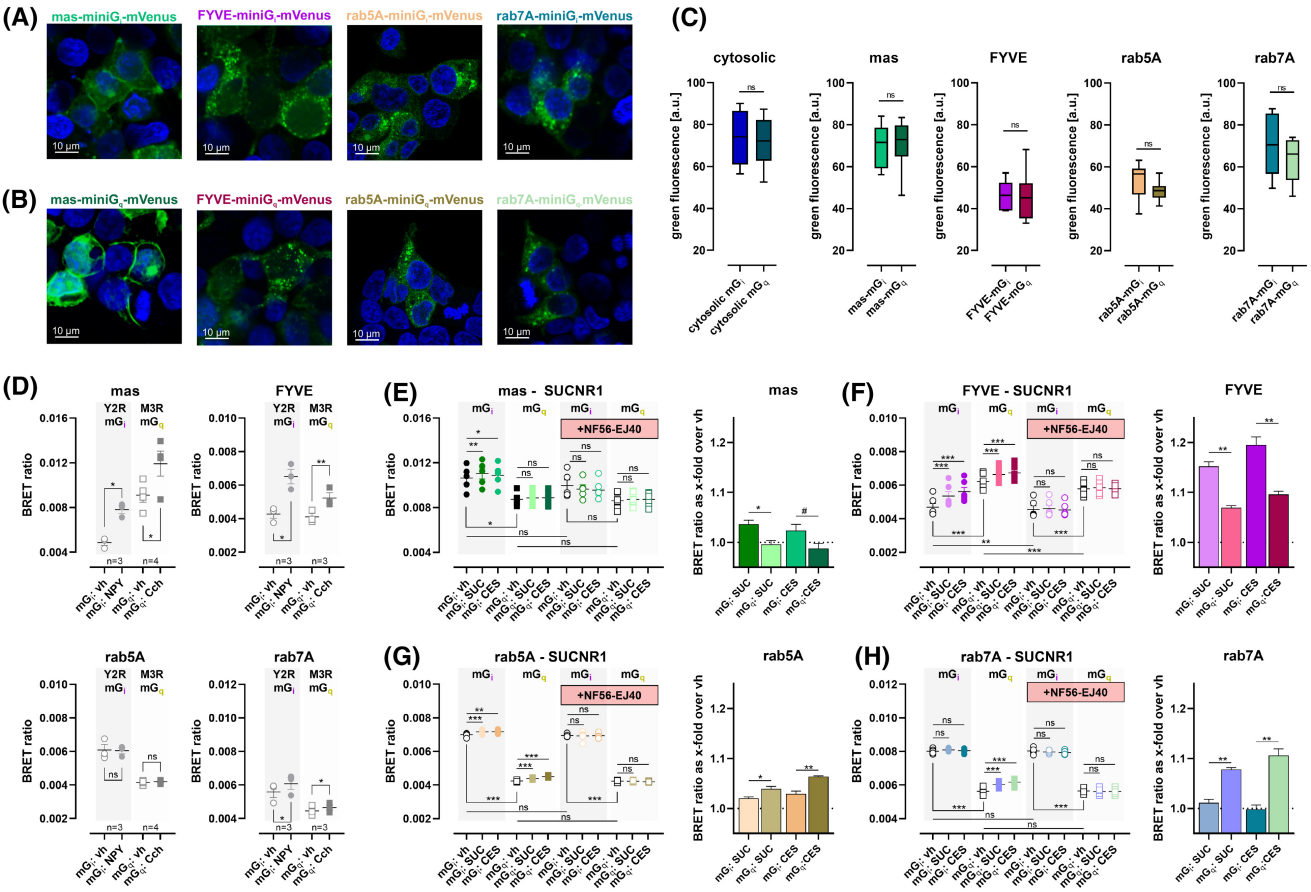
Differential miniG_i_ and miniG_q_ protein recruitment by SUCNR1 dependent on subcellular compartments. For confocal imaging, HEK293‐T cells were transfected with (A) miniG_i_ or (B) miniG_q_ protein‐mVenus variants (green) or (C) both as indicated. Cell nuclei were stained with Hoechst 33342 (blue). (A, B) Representative images of *n* = 3 experiments are shown, and 5 images were taken per experiment. (C) Expression was quantified as green fluorescence using the Celigo Imaging Cytometer (*n* = 3, 3 replicates per experiment). As indicated, BRET assays were performed in cells co‐transfected with (D) Y2R‐Nluc, M3R‐Nluc (number of independent experiments as indicated in the figure), or (E‐H) SUCNR1‐Nluc, and mVenus‐tagged miniG protein variants (all *n* = 6). (D–H) BRET analyses were performed in PBS Glc. (D) Y2R was stimulated with 1 μm neuropeptide Y (NPY) and M3R with 100 μm carbachol (Cch), each serving as a control for G_i_ and G_q_ signaling, respectively. (E–H) SUCNR1 was stimulated with 200 μm succinate (SUC) or *cis*‐epoxysuccinate (CES) with or without preincubation with 100 nm of the SUCNR1 antagonist NF56‐EJ40. (E‐H) BRET ratios before and after agonist stimulation are shown, and for SUCNR1, BRET ratios for SUC and CES are additionally shown as x‐fold over vehicle (vh) control. (E) mas, (F) FYVE, (G) rab5A, and (H) rab7A miniG_i_ and miniG_q_ protein variants. BRET ratios were defined as acceptor emission/donor emission. Data are shown as mean ± SEM. Statistical analyses of BRET ratios were performed using (C) unpaired two‐tailed *t*‐tests, (D) paired two‐tailed *t*‐tests, or (E, F) repeated measures one‐way ANOVA for BRET ratio data (left graph), respectively. (E, F) Statistical analyses of fold‐change BRET (right graph) were performed using unpaired two‐tailed *t*‐tests. ns, not significant; ^#^ *P* ≤ 0.1, *P ≤ 0.05, **P ≤ 0.01, ***P ≤ 0.001.

We used Nluc‐tagged receptors in combination with mVenus‐tagged miniG_i_ and miniG_q_ protein variants that were modified to restrict them to specific subcellular localizations. Adding a membrane anchoring sequence (mas) resulted in plasma membrane localization. Endosomal localization was achieved by fusion to an FYVE domain, rab5A, or rab7A. The particular localization was confirmed using confocal imaging (Fig. 11A,B). No significant differences in expression, which was image‐based quantified as mVenus fluorescence intensity, have been observed comparing miniG_i_ with miniG_q_ in different subcellular compartments (Fig. 11C). Y2R and M3R were always included as controls.

For Y2R and M3R stimulated with their respective agonists, we found a significant increase in BRET ratio for the mas‐, FYVE‐, and rab7A‐ but not the rab5A‐anchored miniG_i_ and miniG_q_ protein variants, respectively (Fig. 11D).

In contrast to the cytosolic and the FYVE‐anchored miniG proteins, the basal BRET ratio of SUCNR1 was significantly lower for the miniG_q_ proteins targeted to the plasma membrane (mas), rab5A, and rab7A than for the miniG_i_ proteins (Figs 7G and 11E–H).

Stimulation of SUCNR1 with SUC or CES resulted in a significant increase in BRET ratio for all miniG_i_ variants except for that anchored in rab7A, while only for mas‐miniG_q_ no increase in BRET ratio upon agonist stimulation was observed (Fig. 11E–H). The presence of NF56‐EJ40 decreased the basal BRET ratio only with the FYVE‐tagged miniG proteins (Fig. 11E–H). However, for all variants, no agonist‐induced increase in BRET ratio with SUCNR1 was detected when cells were pre‐incubated with NF56‐EJ40 (Fig. 11E–H). Finally, we compared the agonist‐induced change in the BRET ratio for miniG_i_ and miniG_q_ targeted to different cellular compartments. While for mas‐ and FYVE‐tagged miniG_i_, as for the cytosolic variants, the agonist‐induced change was higher than that for miniG_q_, the opposite was true for the rab5A‐ and rab7A‐tagged miniG protein variants (Fig. 11E–H).

As a second strategy to analyze localization‐dependent signaling, we used sensors that detect active GTP‐bound Gα proteins (KB1753‐Nluc + Gα_i_‐YFP or GRK2RH‐Nluc + Gα_q_‐mVenus) or free Gβγ subunits (GRK3ct‐Nluc + Gβγ‐mVenus, with Gα_i_ or Gɑ_q_ protein co‐transfected).

The sensors were anchored at the plasma membrane (mas) [42] or localized to endosomes (FYVE, rab7A). KB1753 binds reversibly to GTP‐loaded Gα_i_ but not to Gα_i_‐GDP without affecting intrinsic nucleotide exchange or hydrolysis by Gα_i_. The same is true for GRK2RH (regulator of G protein signaling (RGS) homology (RH) domain of GRK2), which served as the detector module for GTP‐bound Gα_q_. In contrast to the miniG proteins, BRET is not detected between the receptor and G protein but between the respective sensor and the active G protein.

**Fig. 12:**
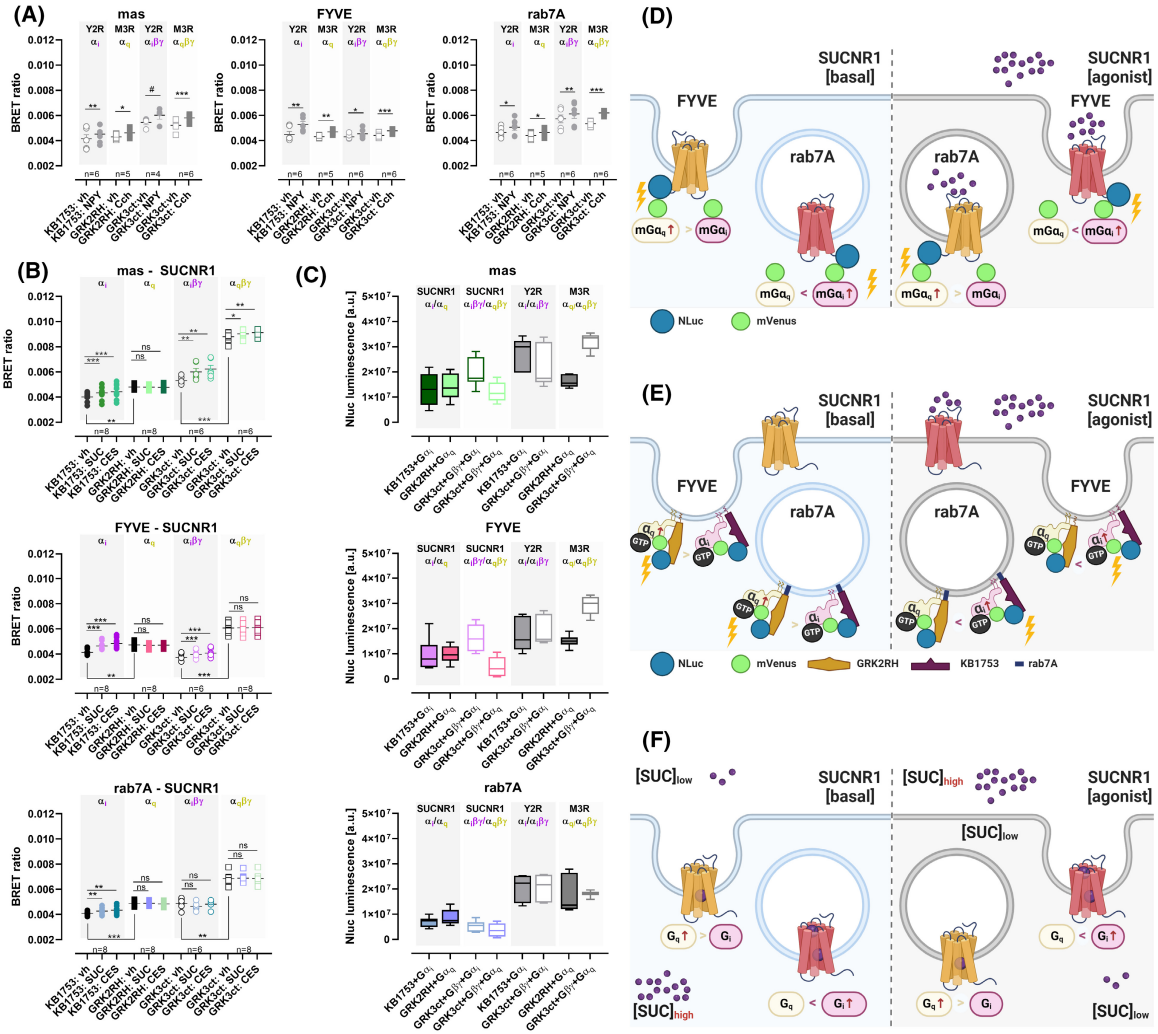
Biosensors for GTP‐bound Gα proteins and free Gβγ proteins in different subcellular compartments. HEK293‐T cells were co‐transfected with (A) Y2R or M3R or (B) SUCNR1 and components constituting compartmentalized biosensors: KB1753‐Nluc and Gα_i_‐YFP or GRK2RH‐Nluc and Gα_q_‐mVenus or GRK3ct‐Nluc, Gβγ‐mVenus and Gα_i_ or GRK3ct‐Nluc, Gβγ‐mVenus and Gα_q_. The Nluc‐tagged biosensors were modified with mas, FYVE, or rab7A. BRET analyses were performed in PBS Glc. (A) Y2R was stimulated with 1 μm neuropeptide Y (NPY). M3R with 100 μm carbachol (Cch). (B) SUCNR1 was stimulated with 200 μm succinate (SUC) or *cis*‐epoxysuccinate (CES). (A, B) BRET ratios before and after agonist stimulation are shown. The number of experiments is indicated in the graphs. (C) Nluc luminescence of the compartmentalized biosensors of the experiments depicted in (A, B) is shown as box and whiskers min to max, line at mean. Statistical analyses were performed using (A) paired two‐tailed *t*‐tests or (B) repeated measures one‐way ANOVA. ns, not significant; ^#^ *P* ≤ 0.1, *P ≤ 0.05, **P ≤ 0.01, ***P ≤ 0.001. (D) Under basal conditions, SUCNR1 in FYVE preferentially recruits miniG_q_, and in rab7A preferentially miniG_i_. When stimulated with an agonist, the opposite is true. (E) Under basal conditions, SUCNR1 leads to more active (GTP‐bound) Gα_q_ than Gα_i_. Agonist stimulation increases active (GTP‐bound) Gα_i_. (F) SUCNR1 has two succinate binding sites [44]. We hypothesize that low SUC concentrations stabilize a G_q_ protein‐activating conformation of SUCNR1 (only one SUC molecule is bound), while high SUC concentrations stabilize a G_i_ protein‐activating SUCNR1 conformation with two SUC molecules bound. Depending on the intra‐ and extracellular SUC levels, SUCNR1 preferentially activates G_q_ or G_i_ proteins from the plasma membrane or endosomes. (D–F) Created with Biorender.com.

For both Y2R and M3R, as controls for G_i_‐ and G_q_‐coupling, we show that stimulation with the agonists increased BRET ratio with the respective sensors (Fig. 12A). For SUCNR1, we found that the BRET ratios in the absence of agonists were significantly higher for mas‐, FYVE‐ and rab7A‐GRK2RH (Gα_q_‐GTP sensor) as compared to the respective KB1753‐(Gα_i‐_GTP) sensor (Fig. 12B). These higher basal values in BRET ratio were also found for Gα_q_‐derived free Gβγ as compared to Gα_i_‐derived free Gβγ subunits (Fig. 12B). Analyses of the basal Nluc luminescence as a measure for the overall expression of a sensor revealed no differences when comparing the sensor for active G_i_ (KB1753) versus active G_q_ (GRK2RH) (Fig. 12C). Stimulation with SUC or CES could increase the BRET ratio of Gα_i_‐GTP in all compartments but not of Gα_q_‐GTP (Fig. 12B). At the plasma membrane and in FYVE‐, but not in rab7A‐endosomes, agonist stimulation of SUCNR1 caused an increase of BRET ratio for G_i_‐derived free Gβγ (Fig. 12B). An increased BRET ratio in G_q_‐derived free Gβγ was only observed at the plasma membrane but not in endosomes (Fig. 12B).

Here, we used the RH domain of GRK2 as a sensor for active Gα_q_. Alternative effectors binding to GTP‐bound Gα_q_ exist, for example, p63RhoGEF [43]. That may potentially explain why we observe an agonist‐induced increase in the BRET ratio for G_q_‐derived free Gβγ but not active GTP‐bound Gα_q_. Further, we showed that SUCNR1 may activate G_q_ protein signaling independent of IP_3_/Ca^2+^ formation (Figs 7 and 8).

In summary, we observed constitutive localization of SUCNR1 at the plasma membrane and in endosomes (Figs 1 and 10A). Under basal conditions, that is, without added agonist, SUCNR1 preferentially recruits cytosolic miniG_q_ and interacts with FYVE‐miniG_q_ (Figs 7G, 11F, and 12D). In contrast, SUCNR1 preferentially interacts with miniG_i_ when anchored to rab5A‐ and rab7A endosomes (Figs 11G,H and 12D). Upon stimulation with SUC or CES, the opposite is true: SUCNR1 preferentially recruits cytosolic, mas‐ and FYVE‐miniG_i_, while in rab5A‐ and rab7A‐endosomes, the interaction of miniG_q_ with SUCNR1 is preferred (Figs 7G, 11E–H, and 12D).

When no agonist is added to SUCNR1, the basal BRET ratio values for active (GTP‐bound) Gα_q_ and G_q_‐derived free Gβγ were higher than those found for G_i_ (Fig. 12B,E). Upon SUCNR1 agonist stimulation, we found stronger G_i_ than G_q_ activation and signaling (Fig. 12B,E). Agonist addition increased GTP‐bound Gα_i_ at the plasma membrane, in FYVE‐ and rab7A‐endosomes, but G_i_‐derived free Gβγ only at the plasma membrane and in FYVE‐endosomes (Fig. 12B,E). Based on the recent findings by Shenol *et al*. [44], proposing that SUCNR1 has two SUC binding sites, we hypothesize that the occupancy of these sites is linked to SUCNR1 signaling. Consequently, binding of SUC to SUCNR1 is tightly linked to local intra‐ and extracellular SUC concentrations and thus cellular metabolic activity (Fig. 12F). We suggest that low SUC concentrations lead to the occupancy of only one SUC binding site in SUCNR1, which stabilizes a G_q_ protein‐activating conformation of SUCNR1 (Fig. 12F). At high SUC concentrations, both SUC binding sites of SUCNR1 are occupied, which stabilizes the G_i_ protein‐activating conformation (Fig. 12F). Since SUC concentrations may be higher either extra‐ or intracellularly, depending on the cellular metabolic rate, available energy substrates, and local extracellular changes, SUCNR1 may have bound one or two SUC molecules either when residing at the plasma membrane or in endosomal compartments (Fig. 12F). The diminished G_q_ signaling in Gln is the consequence of the increased extracellular SUC concentrations that cause increased internalization and predominant G_i_ signaling (Figs 2B and 7, 8, 9, 10).

### SUCNR1 is functionally expressed in M1‐ and M2‐like macrophages and regulates cellular metabolism

**Fig. 13:**
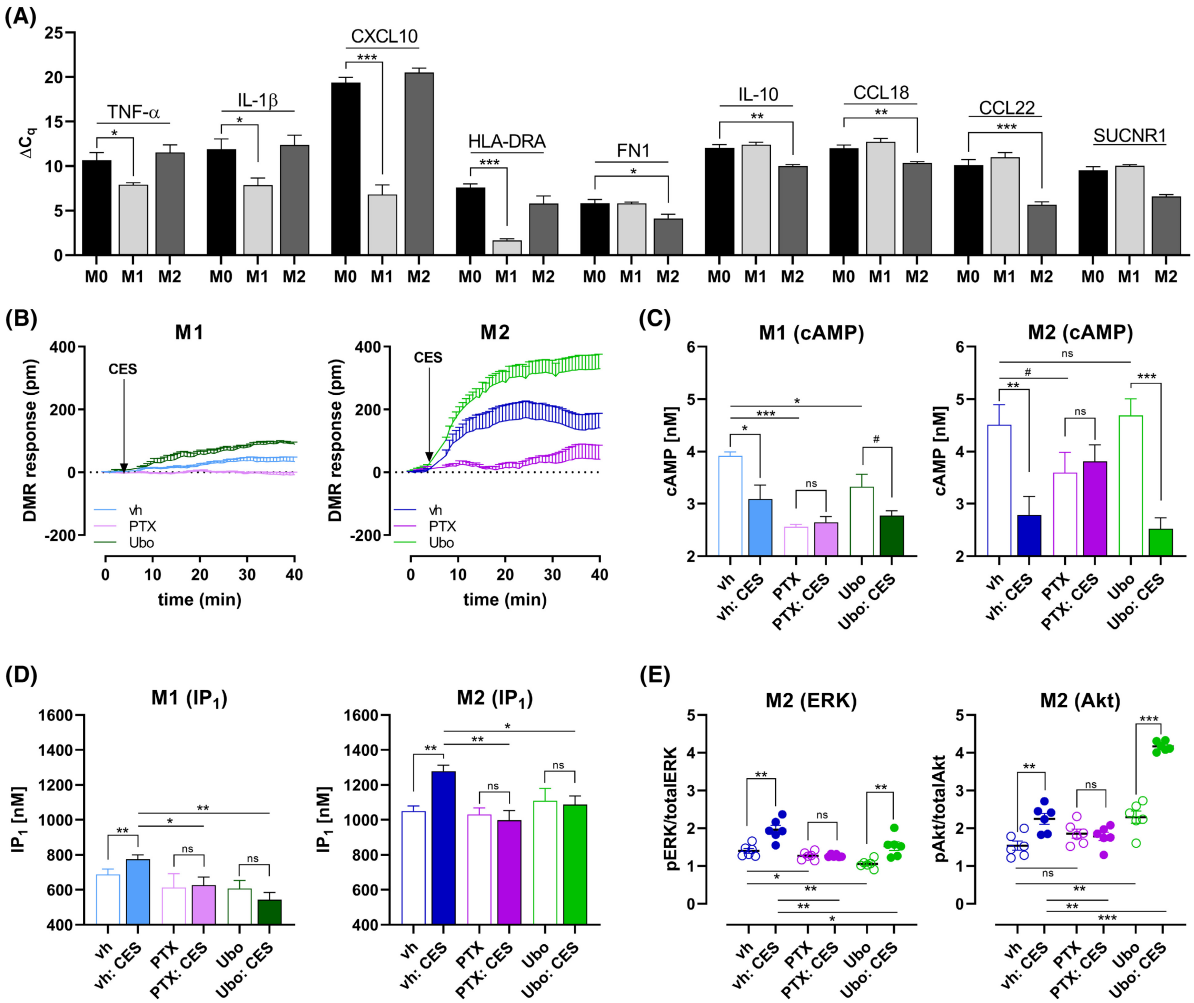
THP‐1‐derived M1‐ and M2‐like macrophages functionally express SUCNR1. (A) The mRNA expression levels of markers for M1‐ and M2‐like macrophages and SUCNR1 are shown as Δ*C*_q_‐values for THP‐1 cells differentiated to M0‐, M1‐, or M2‐like macrophages (*n* = 3, reference gene ACTB *C*_q_ = 16). (B) Dynamic mass redistribution (DMR) responses in M1‐ and M2‐like macrophages upon stimulation with 200 μm *cis*‐epoxysuccinate (CES) with or without pertussis toxin (PTX, 100 ng·mL^−1^, 16 h), or UBO‐QIC (Ubo, 300 nm, 30 min) pre‐incubation (*n* = 3). (C) cAMP inhibitory signaling (M1: *n* = 3, M2: *n* = 5) (D) and intracellular IP_1_ levels (*n* = 3) in response to 200 μm *cis*‐epoxysuccinate (CES) with or without pertussis toxin (PTX, 100 ng·mL^−1^, 16 h) or UBO‐QIC (Ubo, 300 nm, 30 min) pre‐incubation. (E) Phosphorylation of endogenous ERK (*n* = 6) and Akt1/2/3 (*n* = 6) in cellular lysates of M2‐like macrophages stimulated with 200 μm CES with or without pertussis toxin (PTX, 100 ng·mL^−1^, 16 h) or UBO‐QIC (Ubo, 300 nm, 30 min) pre‐incubation. (A–E) Data are shown as mean ± SEM. Statistical analyses were performed using (A) unpaired two‐tailed *t*‐tests or (C–E) paired two‐tailed *t*‐tests (comparing unstimulated versus CES for each condition) or repeated measures one‐way ANOVA (comparing vh versus PTX and Ubo‐treated), respectively. ns, not significant; ^#^ *P* ≤ 0.1, *P ≤ 0.05, **P ≤ 0.01, ***P ≤ 0.001.

Previous research has shown that macrophages express SUCNR1 [45, 46]. We differentiated the human monocytic THP‐1 (Tohoku Hospital Pediatrics‐1) cell line into M0‐, M1‐, or M2‐like macrophages as described before in detail [47]. This study also performed an exhaustive analysis of specific macrophage markers for the chosen polarization conditions [47]. Here, we analyzed only selected markers specific for M1‐ and M2‐like macrophages to check whether we observed the previously described pattern when applying the same protocol. Indeed, the pro‐inflammatory cytokines tumor necrosis factor α (TNF‐α), interleukin‐1β (IL‐1β), and C‐X‐C motif chemokine ligand 10 (CXCL10) are classical M1 markers and exhibited a significantly increased mRNA expression in THP‐1‐derived M1‐like macrophages, as did the membrane receptor major histocompatibility complex, class II, DRα (HLA‐DRA) (Fig. 13A). THP‐1‐derived M2‐like macrophages showed an increased mRNA expression of classical M2 markers, that is, fibronectin (FN1), interleukin 10 (IL‐10), C–C motif chemokine ligand 18 (CCL18), and CCL22 as shown before [47] (Fig. 13A).

The highest SUCNR1 mRNA expression was found in M2‐like macrophages, but M0‐ and M1‐like macrophages also exhibited considerable SUCNR1 expression (Fig. 13A). DMR analyses in both types of macrophages showed that the detectable CES‐induced response was much more robust in M2‐like macrophages, likely due to the higher SUCNR1 expression (Fig. 13B). This response was diminished by the G_i_ inhibitor PTX and increased when cells were pre‐incubated with the G_q_ inhibitor Ubo, showing that the CES‐induced signal is a sum of both G_i_ and G_q_‐mediated responses (Fig. 13B). Using cAMP inhibition assays, we found a PTX‐sensitive but Ubo‐insensitive decrease in cAMP levels upon stimulation with CES in both M1‐ and M2‐like macrophages (Fig. 13C). In IP_1_ accumulation assays, the detected CES‐induced increase in IP_1_ levels was sensitive to PTX and Ubo, indicating that G_i_ protein activation is also required for G_q_ protein signaling (Fig. 13D). That was also observed in HEK293‐T cells, where Ca^2+^ release was also diminished in the presence of PTX (Fig. 8A). All these results indicate the functional presence of SUCNR1 in both types of macrophages, with the recorded responses reflecting the differences in SUCNR1 mRNA and, thus, protein abundance. To further understand the obtained DMR responses in the presence of PTX and Ubo, we analyzed CES‐induced ERK and Akt signaling in M2‐like macrophages (Fig. 13E). As observed in SUCNR1‐expressing HEK293‐T cells, stimulation with CES resulted in a significant increase in both ERK and Akt phosphorylation, which was inhibited by PTX, indicating that it is G_i_ protein‐mediated (Figs 9A,B and 13E). Pre‐treatment with the G_q_ inhibitor Ubo caused a small but significant decrease in ERK and an increase in Akt signaling in the absence of an agonist (Fig. 13E). This phenomenon was also observed in SUCNR1‐expressing HEK293‐T cells (Fig. 9A,B). Application of CES clearly increased Akt phosphorylation in Ubo‐treated cells (Fig. 13E). In summary, this supports a G_q_‐mediated limitation of SUCNR1‐specific G_i_‐induced Akt activation. ERK, on the other hand, appears to be mainly activated through G_i_ protein upon SUCNR1 activation.

**Fig. 14:**
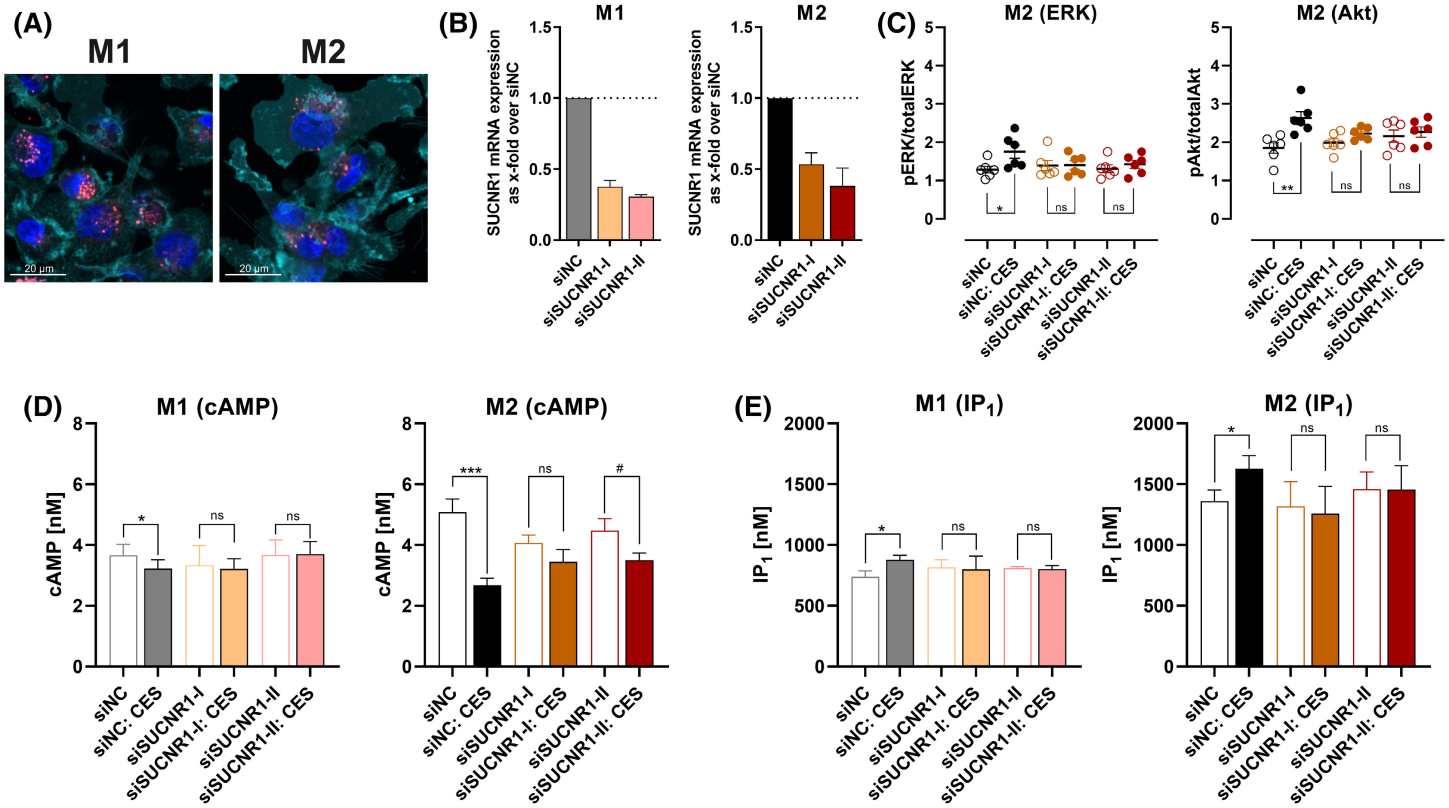
THP‐1‐derived M1 and M2‐like macrophages exhibited SUCNR1‐specific G_i_‐ and G_q_ signaling. (A) M1‐ and M2‐like macrophages were transfected with a red‐fluorescent siRNA, stained Hoechst 33342 (blue), and CellMask plasma membrane stain (cyan) for confocal imaging. Representative images of *n* = 3 experiments are shown. (B) THP‐1‐derived M1‐ or M2‐macrophages were transfected with two different SUCNR1‐specific siRNAs or siNC (negative Ctrl siRNA), which caused at least ~ 50% reduction in SUCNR1 mRNA levels as detected using RT‐qPCR. (*n* = 3, reference gene ACTB *C*_q_ = 18.3) (C) Phosphorylation of endogenous ERK (*n* = 6) and AKT1/2/3 (*n* = 6) in cellular lysates of M2‐like macrophages stimulated with 200 μm CES with (siSUCNR1‐I/‐II) or without (siNC) knock‐down of SUCNR1. (D) cAMP inhibitory signaling (M1: *n* = 3, M2: *n* = 5) and (E) intracellular IP_1_ levels (*n* = 3) in response to 200 μm *cis*‐epoxysuccinate (CES) with (siSUCNR1‐I/‐II) or without (siNC) knock‐down of SUCNR1. Data are shown as mean ± SEM. Statistical analyses were performed by applying paired two‐tailed *t*‐tests. ns, not significant; ^#^ *P* ≤ 0.1, *P ≤ 0.05, **P ≤ 0.01, ***P ≤ 0.001.

Next, we established siRNA transfection of THP‐1‐derived M1‐ and M2‐like macrophages using fluorescently labeled siRNA (Fig. 14A). To determine receptor‐specific effects, we transfected THP‐1‐derived M1‐ and M2‐like macrophages with two different siRNAs targeting SUCNR1. Successful knockdown was confirmed using RT‐qPCR, which showed a reduction of SUCNR1 mRNA expression by 50–70% (Fig. 14B). Knock‐down efficiency on protein level in THP‐1 cells could not be determined due to the lack of specific antibodies. However, we have previously shown in heterologously SUCNR1‐expressing HEK293‐T cells that the chosen siRNAs indeed cause a reduction in SUCNR1 protein expression [23]. In addition, all second messenger analyses, that is, cAMP, IP_1_, as well as ERK and Akt phosphorylation, showed that the CES‐induced signals were abolished or at least diminished in cells transfected with siRNA specifically directed against SUCNR1 (Fig. 14C–E).

**Fig. 15:**
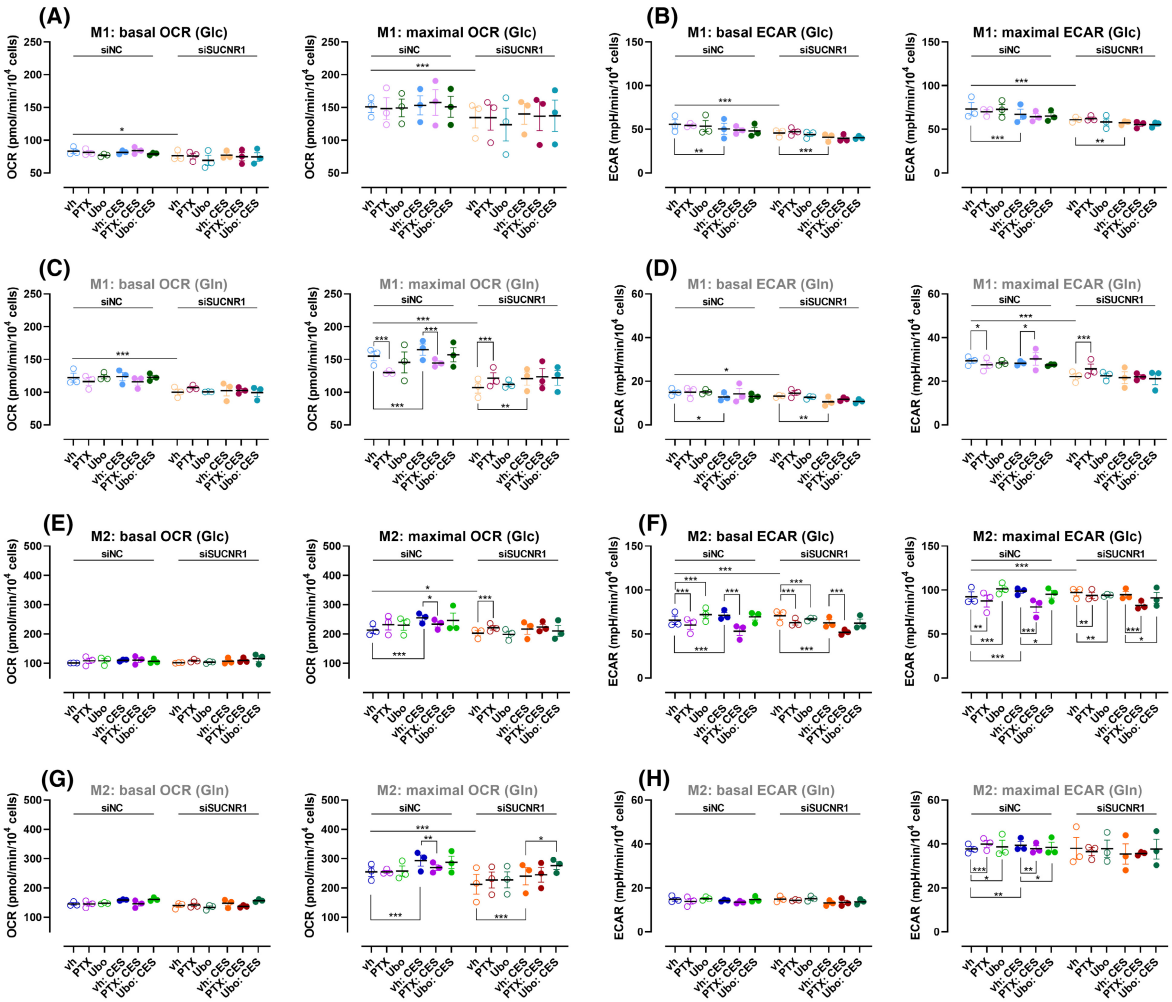
SUCNR1 in THP‐1‐derived M1‐ and M2‐like macrophages regulates cellular metabolism. Oxygen consumption rate (OCR) and extracellular acidification rate (ECAR) were measured with the Seahorse XFe96 extracellular flux analyzer in the presence of 10 mm glucose (Glc) or 2 mm glutamine (Gln), stimulated with 200 μm *cis*‐epoxysuccinate (CES) and pre‐incubated with or without pertussis toxin (PTX, 100 ng·mL^−1^, 16 h), or UBO‐QIC (Ubo, 300 nm, 30 min). (A) THP‐1‐M1: basal and maximal OCR in Glc. (B) THP‐1‐M1: basal and maximal ECAR in Glc. (C) THP‐1‐M1: basal and maximal OCR in Gln. (D) THP‐1‐M1: basal and maximal ECAR in Gln. (E) THP‐1‐M2: basal and maximal OCR in Glc. (F) THP‐1‐M2: basal and maximal ECAR in Glc. (G) THP‐1‐M2: basal and maximal OCR in Gln. (H) THP‐1‐M2: basal and maximal ECAR in Gln. OCR and ECAR are depicted as mean ± SEM of *n* = 3 independent experiments, each carried out in six technical replicates. Statistical analyses were performed using a two‐way repeated measures ANOVA. *P ≤ 0.05, **P ≤ 0.01, ***P ≤ 0.001.

Depending on their polarization state, these macrophages exhibit characteristic metabolic phenotypes. M1‐like macrophages are characterized by a high glycolytic rate, while M2‐like macrophages exhibit increased mitochondrial respiration [48, 49]. We performed the Seahorse XF Cell Mito Stress Test in the presence of Glc or Gln, stimulated with CES, and used PTX and Ubo to evaluate the contribution of the respective signaling pathways to the metabolic effects (Fig. 15). To determine SUCNR1‐specificity, we employed siRNA‐mediated knockdown (Fig. 15).

In M1‐like macrophages, the knockdown of SUCNR1 was always accompanied by a reduction of both OCR and ECAR independently of the available energy substrate (Fig. 15A–D). ECAR, with Gln as the sole energy substrate present, reflects bicarbonate production during respiration and, thus, non‐glycolytic acidification. With Glc as the available energy substrate, ECAR mainly reflects glycolytic rate, that is, conversion of Glc to pyruvate, metabolized to lactate and protons, which are then exported out of the cell. In the Gln medium, inhibition of G_i_ by PTX caused a decrease in maximal OCR in siNC‐transfected THP‐1‐M1, while the opposite was true in siSUCNR1‐transfected cells (Fig. 15C). That was also observed for maximal ECAR, which in Gln, that is, in the absence of Glc, primarily reflects OCR (Fig. 15D). Stimulation with CES in Gln caused an increase in maximal OCR and a decrease in basal ECAR, independent of SUCNR1 (Fig. 15C,D).

In M2‐like macrophages, the knockdown of SUCNR1 caused a decrease in maximal OCR in Glc and Gln (Fig. 15E–G). Stimulation with CES resulted in a SUCNR1‐specific, PTX‐sensitive increase in maximal OCR (Fig. 15E,G). Moreover, CES‐stimulated M2‐like macrophages showed a SUCNR1‐specific increase in ECAR (Fig. 15F,H). In the Glc‐containing medium (no CES), the inhibition of G_q_ with Ubo led to an increase in glycolytic rate (ECAR) in siNC‐transfected cells, while the opposite was true in siSUCNR1‐transfected cells (Fig. 15F). This suggests that under unstimulated conditions, SUCNR1‐mediated G_i_ activation is associated with an increase in glycolytic activity. Basal G_q_ activation, on the other hand, limits glycolysis, providing a reasonable explanation for the observed effects. However, in Gln, both PTX and Ubo cause an increase in maximal ECAR, which primarily reflects the oxidative phosphorylation (OXPHOS) rate. Under these conditions, SUCNR1 basally inhibits the respiratory chain through G_i_ and G_q_.

**Fig. 16:**
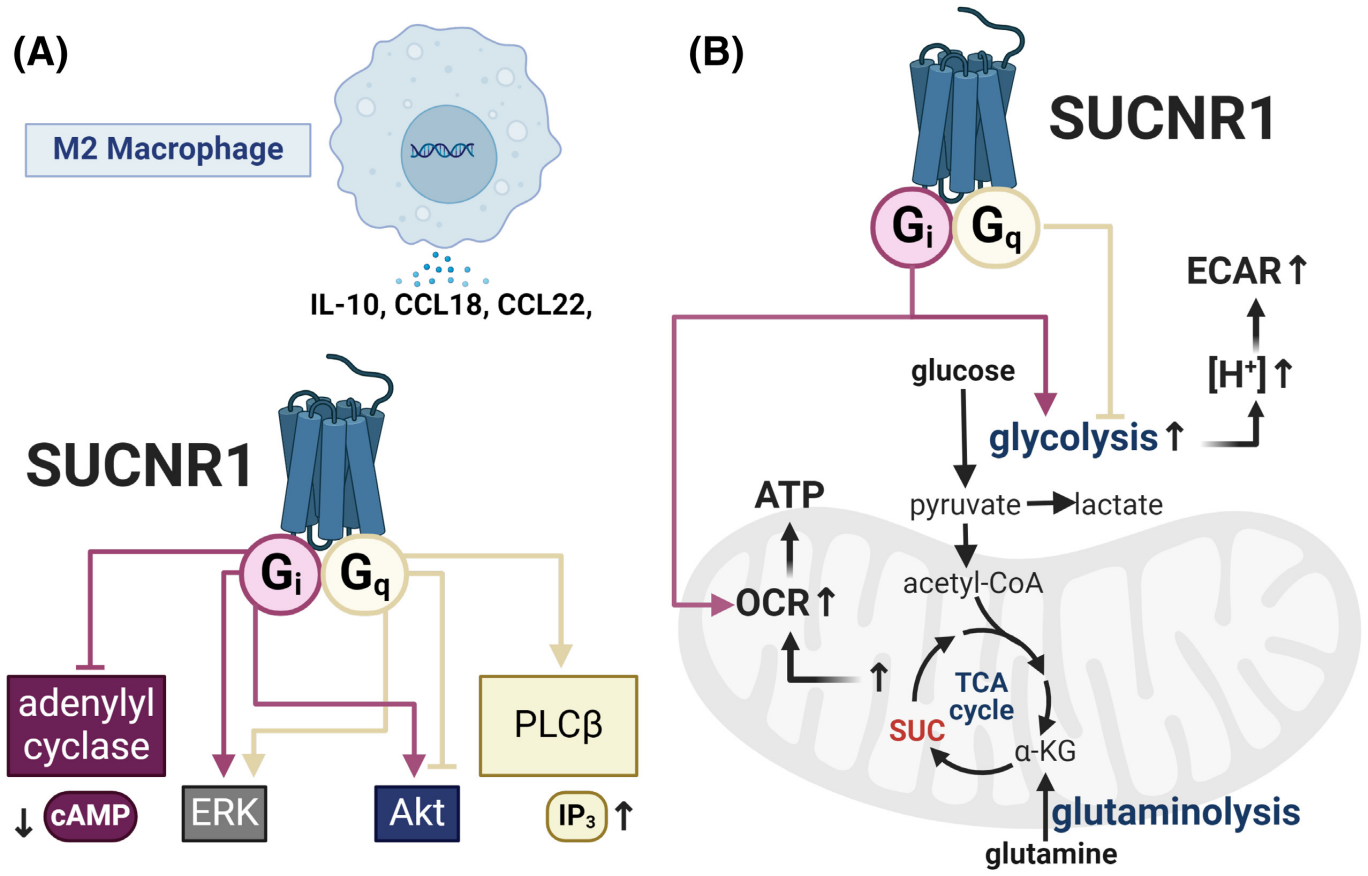
Graphical summary of findings on SUCNR1 signal transduction and effect on metabolism in THP‐1‐derived M2‐like macrophages. (A) M2‐like macrophages functionally express SUCNR1, a G_i_‐ and G_q_‐coupled receptor that activates ERK and Akt phosphorylation through G_i_ and inhibits Akt phosphorylation through G_q_. (B) SUCNR1 stimulates oxidative phosphorylation (OCR) and glycolytic rate (ECAR) through G_i_ protein. SUCNR1 inhibits glycolytic rate (ECAR) through G_q_ protein. Created with Biorender.com.

In summary, we find that SUCNR1 is functionally expressed in THP‐1‐derived M2‐like macrophages, which are characterized by an increased expression of the cytokines IL‐10, CCL18, and CCL‐22 (Fig. 16A). In these cells, SUCNR1 activates ERK and Akt phosphorylation through G_i_ protein activation and inhibits Akt phosphorylation through the G_q_ protein (Fig. 16A). SUCNR1 activation increases OXPHOS and glycolytic rate primarily through G_i_ protein activation, while activation of G_q_ protein reduces the glycolytic rate (Fig. 16B).

## Discussion

Elevated SUC levels occur in certain physiological conditions, such as endurance exercise and pathologies, including hypertension, type 2 diabetes mellitus, or obesity. Thus, intra‐ and extracellular SUC concentrations may be high enough to activate SUCNR1, depending on the cellular context and metabolic state [5]. SUCNR1 is associated with pro‐ and anti‐inflammatory effects, and only a few reports have confirmed its G_q_‐ besides G_i_‐coupling [13, 14, 21, 22]. Here, we show that SUCNR1 exhibits high basal G_q_ protein activation depending on cellular metabolism, which may hamper the detection of a response upon agonist addition.

Due to its observed contradictory role in inflammation, SUCNR1 has gained attention as a potential drug target, and synthetic ligands have been developed. Efforts to design SUCNR1 non‐metabolite agonists and antagonists yielded compound **31** as an agonist and NF56‐EJ40 as a human SUCNR1‐specific antagonist.

Our analyses of the signaling properties of compound **31** revealed that it exhibits a comparable potency to CES at SUCNR1 in cAMP inhibition assays of about 50 nm (Fig. 4B). However, at 100 μm, compound **31** failed to induce G_q_ activation and arrestin‐3 recruitment (Fig. 4C–F). Together, our results suggest that compound **31** is a partial SUCNR1 agonist (Fig. 4). The human‐specific SUCNR1 antagonist NF56‐EJ40 increased SUCNR1 presence at the plasma membrane and recruitment of arrestin‐3 upon agonist stimulation (Fig. 5F,G). In addition, G_q_ ‐and G_i_‐mediated SUCNR1 signaling was inhibited by NF56‐EJ40, which is reflected in the abolished IP_1_/Ca^2+^ and cAMP inhibitory signaling, respectively (Fig. 5A–E). The recruitment of miniG_i_ and miniG_q_ protein upon agonist stimulation was entirely abolished in the presence of NF56‐EJ40 (Figs 7G and 11E–H). Future studies using those non‐metabolite compounds acting at SUCNR1 should consider those aspects.

Our research aimed to link cellular metabolism to SUCNR1 localization and signaling.

We found that SUCNR1 affects mitochondrial respiration in HEK293‐T cells, heterologously overexpressing SUCNR1 and in endogenously SUCNR1 expressing THP‐1‐derived macrophages (Figs 6 and 15). Cellular metabolic state and intra‐ and extracellular SUC concentration changes are tightly linked and dependent on the available energy substrates (Fig. 2B). This is, at least partially, regulated by SUCNR1, but it also affects the receptor's localization and signaling (G_i_ and G_q_ protein activation).

Given the complexity of this system involving SUC metabolism and transport, its extra‐ versus intracellular origin, the variable subcellular localization of SUCNR1, and its various activated signaling pathways, we initially chose a simplified model. HEK293‐T cells that heterologously express SUCNR1 were incubated in a medium containing either Glc or Gln to mimic different metabolic states.

Glc is metabolized to pyruvate via glycolysis, which enters the TCA cycle or can be converted to lactate (Fig. 2A). High glycolytic rates result in elevated proton concentrations, indicated by the ECAR (Figs 10F, 15, and 16B). With Glc as the only available energy substrate, more SUC was detected intracellularly, and the overall OCR was lower than that observed with Gln (Figs 2B, 6A, and 15).

Conversely, Gln fuels the TCA cycle through glutaminolysis, associated with a lower ECAR reflecting a reduced extracellular proton concentration (Figs 2A, 6A, and 15) [50]. An exceedingly high OCR is linked to a high mitochondrial membrane potential [51]. This may contribute to intracellular SUC accumulation, which may be released from the cells through transporters belonging to the SLC13 and MCT families (Fig. 2B,C).

MCT1 and MCT4 have been described as potential SUC transporters [52, 53]. In HEK293‐T cells, MCT4 mRNA levels were down‐regulated in Glc, whereas intracellular SUC levels were increased, suggesting its involvement in SUC export. On the other hand, SUC uptake may be mediated through a Na^+^‐dependent transporter of the SLC13 family, like SLC13A4, a transporter expressed in kidney cells and known to transport SUC [54, 55]. Upregulation of mRNA levels in Glc and SUCNR1‐expressing cells suggests its involvement in increased intracellular SUC levels (Fig. 2B,C). However, future studies linking SUCNR1 and SUC transporter function on protein levels in different cellular systems will be needed to shed further light on this connection.

In our system, SUC concentrations ranged intracellularly from ~ 7 μm in Glc to ~ 14 μm in Gln, and we detected up to ~ 2.3 μm extracellularly in Gln (Fig. 2B). In Gln, we found SUCNR1, to a greater extent, co‐localized with endosomal marker proteins caused by increased receptor internalization, likely due to the elevated extracellular SUC concentrations (Fig. 10C). Gln alone induced receptor interaction with arrestin‐3 (Fig. 8I). Moreover, SUCNR1‐mediated G_i_‐signaling was enhanced in Gln while G_q_ signaling was dampened (Fig. 10F). That was reflected in lower DMR signals and clearly decreased Ca^2+^ signals (Figs 7C and 8D,E). Using the GLS1 inhibitor BPTES, SUCNR1‐mediated Ca^2+^ signaling in Gln could be partially rescued, further establishing the link between Gln metabolism and G_q_ signaling (Fig. 8F). Hence, SUCNR1 is increasingly located intracellularly in Gln, causing reduced G_q_ signaling and more pronounced G_i_ signaling. SUCNR1 was found at the plasma membrane and co‐localized besides the endosomal marker proteins rab5A, rab7A, and rab11A with clathrin and caveolin (Figs 1B,E,G and 10A). Mature caveolae formation also requires PIP_2_, and caveolin may facilitate caveolae localization without triggering receptor internalization (reviewed in Ref. [56]). Caveolin can also interact with Gα_q_ subunits but not Gβγ or other Gα proteins [57, 58]. That suggests a potential involvement of caveolin in SUCNR1‐mediated G_q_ signaling. The observed SUCNR1‐dependent Ca^2+^ signal and ERK phosphorylation depended on active G_i_ and G_q_ (Figs 8A–C and 9A) [59]. Ubo caused an increase in basal Akt, suggesting that G_q_ inhibits Akt signaling SUCNR1‐specific (Figs 9B and 13E). PTX diminished agonist‐induced Akt phosphorylation below basal levels (Fig. 9B). We conclude that SUCNR1‐mediated Akt activation may be inhibited through G_q_ protein‐dependent inhibition of G_i_ protein.

Despite the almost complete absence of Ca^2+^ signals in Gln, we found not only G_i_‐ but also G_q_‐dependent signaling upon SUCNR1 stimulation with SUC and CES (Figs 7 and 8D). In recent years, it has become clear that G protein signaling may originate from both the plasma membrane and endosomes [60].

For SUCNR1, we found constitutive localization of SUCNR1 in endosomes associated with higher basal active (GTP‐bound) Gα_q_ than Gα_i_ protein (Figs 1, 7G, 10A, and 12B). SUCNR1 agonist stimulation induced a stronger G_i_ than G_q_ signaling that persisted partially from the plasma membrane into endosomes (Fig. 12). In rab7A endosomes under basal conditions, SUCNR1 showed stronger recruitment of miniG_i_, while we found preferential miniG_q_ recruitment upon agonist stimulation (Figs 7G, 11E–H, and 12D). Shenol *et al*. [44] recently proposed that SUCNR1 has two distinct SUC binding sites. Based on these findings, we hypothesize that the occupancy of these two sites is linked to SUCNR1 signaling, local intra‐ and extracellular SUC concentrations, and thus cellular metabolism (Fig. 12F). At low SUC concentrations, only one SUC binding site is occupied in SUCNR1, which stabilizes a G_q_ protein‐activating conformation (Fig. 12F). If SUC concentrations are high, two SUC molecules are bound at SUCNR1, thereby stabilizing a conformation that preferentially activates G_i_ proteins (Fig. 12F). SUC concentrations, in turn, may be high intra‐ and/or extracellularly, as shown in our simplified experimental setup using Glc or Gln as the only available energy substrate (Fig. 2B). Physiologically, this may be caused by changes in the metabolic activity of different cell types or tissues and associated with processes like inflammation. We suggest that SUCNR1 serves as a sensor for SUC both intra‐ and extracellularly, thus functioning in an auto‐ and paracrine manner.

Plasma membrane‐located Gα_q_, PIP_2_, and caveolin are necessary for IP_3_ formation and, thus, Ca^2+^ release from the ER. Multiple publications demonstrated a link between Ca^2+^ release from the ER and subsequent ATP increase in the mitochondria (reviewed in Refs [61, 62]). This aligns with our findings that extracellular SUC or CES addition in the Glc‐containing medium caused an increase in basal and maximal respiration (Fig. 6B). This may lead to a significant accumulation of reactive oxygen species (ROS) and cell death (reviewed in Ref. [63]). Thus, in conditions associated with high OXPHOS, reflected in our experimental setup with Gln as the only available energy substrate, Ca^2+^ release from the ER through SUCNR1 activation was reduced (Fig. 8A,D,E), and extracellular addition of SUC or CES caused a reduction in maximal respiration (Fig. 6C). We suggest that SUCNR1 acts as a control system that balances the rate of cellular energy production, depending on the cellular metabolic state, to avoid an extensive accumulation of ROS. Our previous findings that SUCNR1 knockdown increases mitochondrial respiration and ROS in Gln‐dependent cancer cells support this conclusion [23].

THP‐1‐derived macrophages served as a model for endogenously SUCNR1‐expressing immune cells. Certain metabolic traits characterize pro‐inflammatory M1‐like and anti‐inflammatory M2‐like macrophages. M1‐like macrophages typically rely more on glycolysis, whereas M2‐like macrophages display a more aerobic phenotype relying on OXPHOS. We confirmed that in our metabolic analyses (Fig. 15). Both metabolic parameters, OCR and ECAR, are differentially regulated in M1‐ and M2‐like macrophages, depending on the metabolic context, the presence of SUCNR1, and stimulation with agonist (Fig. 15). However, in M2‐like macrophages with Glc available, basal SUCNR1‐mediated G_i_ activation results in increased glycolytic activity, while basal G_q_ activation limits glycolysis (Figs 15 and 16B). In Gln, mimicking a high metabolic rate characterized by a high rate of OXPHOS, SUCNR1 inhibits the respiratory chain through G_i_ and G_q_ (Figs 15 and 16B). These findings further argue for the relevance of SUCNR1 in cellular energy homeostasis and the differential regulation of its signaling depending on the metabolic state.

The accumulation of SUC in macrophages is associated with a low extracellular pH and is known to activate a pro‐inflammatory program and the inflammasome [45, 64, 65]. Moreover, chronic activation of macrophages causes an increase in intracellular SUC via glutamine‐dependent anaplerosis and the “GABA‐shunt” pathway. There, SUC acts as an endogenous danger signal to regulate pro‐inflammation [66]. Previous studies reported that SUC may act as a pro‐ and/or anti‐inflammatory metabolite and SUCNR1 as a para‐ and autocrine sensor of SUC concentrations [1, 67]. Our findings, for example, the differential G_q_‐regulated ERK and Akt signaling (Figs 13E and 16B), may help to understand SUCNR1 signaling in the context of pro‐ and anti‐inflammatory macrophages, depending on the metabolic context. However, the physiological relevance of these signaling pathways, that is, for cell proliferation or secretion of interleukins modulating the inflammatory response, remains to be elucidated.

Putting our findings in a broader context, we hypothesize that they provide a reasonable basis for explaining some of the contradictory observations on the role of SUCNR1 in inflammation. Many studies have focused on SUCNR1 function in both adipocytes and macrophages in the context of obesity. In adipocytes, SUCNR1 is, besides its anti‐lipolytic role, responsible for intact glucose homeostasis and prevents metabolic consequences of diet‐induced obesity. Adipocyte G_q_ signaling represents a key regulator of glucose homeostasis and peripheral insulin sensitivity under physiological and pathophysiological conditions [68]. SUCNR1‐mediated G_q_ signaling may be diminished in adipocytes from obese patients partially due to the metabolic mechanisms described above for our simplified model. In addition, SUCNR1 potentiates insulin secretion and has recently been identified as a regulator of leptin signaling, thereby mediating nutrient‐related leptin dynamics to control whole‐body homeostasis [69, 70]. Keiran *et al*. [45] showed that Sucnr1‐deficient mouse adipose tissue macrophages (ATMs) from subcutaneous fat adopt a pro‐inflammatory phenotype, while Sucnr1‐deficient ATMs from visceral fat show a dampened inflammatory status. They also found that SUC decreased the expression of inflammatory markers in adipose tissue from lean human subjects but not from obese subjects [45]. Harber *et al*. [12] show that a macrophage SUCNR1 deficiency caused an enhanced inflammatory response without the addition of exogenous SUC. They suggested that the inflammatory effects of SUC and SUCNR1 in macrophages are context‐dependent. We hypothesize that this effect is at least partially regulated via Akt, as this is known to reduce inflammation [71]. G_q_ might additionally be involved as a regulator to prevent overly excessive Akt activation and balance anti‐inflammatory responses in M2 macrophages (Figs 13E and 16B).

Overall, we propose that the metabolic state and intra‐/extracellular pH must be considered in the experimental setup and when interpreting experiments regarding SUC and SUCNR1. A “high energy” metabolic state may, for example, be induced by hyperglycemia, which has previously been shown to drive the release of SUC in adipose tissue [1]. In cell culture conditions, this may be mimicked using a high Glc (25 mm) and Gln (4 mm)‐containing medium (i.e., DMEM). Our findings suggest a mechanism of SUCNR1 signaling underlying its opposing roles in inflammation: Under physiological conditions without acute inflammation, macrophage metabolism is balanced without SUC accumulation and an extracellular pH of around 7.4. SUCNR1 is primarily present at the plasma membrane and signals via G_i_ and G_q_, mediating anti‐inflammatory responses. Pro‐inflammatory signals, for example, acidification of the extracellular space, promote SUC import, with that intracellular signaling of SUCNR1 and reduce G_q_ signaling from the plasma membrane. SUCNR1 switches its function to support pro‐inflammatory signaling. How this affects M1 and M2 polarization and activation of macrophages, including, for example, secretion of interleukins, needs to be established in future analyses.

Similar mechanisms might be valid for SUCNR1 signaling in adipose tissue. In healthy adipose tissue, SUC is not accumulated or exported, and circulating SUC levels are low. SUCNR1 adipocyte signaling via G_i_ and G_q_ maintains glucose homeostasis. Obesity is associated with impaired glucose metabolism, leading to elevated levels of circulating SUC and concomitant SUC accumulation [70, 72]. In addition, chronic inflammation in adipose tissue is observed in obesity, accompanied by a decrease in extracellular pH. This may cause a reduction of SUCNR1 at the plasma membrane and increase intracellular versus extracellular SUCNR1 signaling, which may partially be responsible for impaired glucose homeostasis in adipocytes.

We suggest that SUCNR1 functions as an auto‐ and paracrine sensor that mediates pro‐ or anti‐inflammatory responses, depending on several factors. These include the metabolic activity of SUCNR1‐expressing and surrounding cells, intra‐ and extracellular SUC levels, the availability of energy substrates, and pH. These factors are responsible for differential SUCNR1 signaling outcomes. The metabolite SUC integrates bioenergetic parameters of oxygen availability, substrate supply, ATP utilization, and intracellular pH to some extent through SUCNR1 [51]. Effects like SUCNR1‐mediated inhibition of lipolysis in adipocytes [73] or induction of cell migration in immune cells [1] are likely mediated through G_i_ activation. However, metabolic reprogramming of M2 macrophages or browning of white adipose tissue may be G_q_‐mediated. Therefore, signaling compounds that activate SUCNR1‐induced G_q_‐signaling may be required to obtain a particularly desired pharmacological effect. SUCNR1 still represents an attractive therapeutic strategy to modulate macrophage responses and inflammation in several pathological contexts.

Our work provides substantial evidence that the exploitation of SUCNR1 as a drug target needs to carefully consider the metabolic context and the fact that the actual agonist SUC is constantly produced by cells and microbiota under both physiological and pathophysiological conditions. Other TCA metabolites, for example, oxaloacetate and itaconate, also accumulate under conditions of stress and hypoxia and have the potential to activate SUCNR1 (Fig. 3). SUCNR1 activation by these metabolites and the activation of G_i_ and G_q_ from different cellular compartments further increase this complexity. This localization‐ and metabolism‐dependent signaling of SUCNR1 must be considered for further drug development targeting SUCNR1. Our results provide a first step toward understanding the divergent outcomes of acute versus chronic SUC‐SUCNR1 signaling and provide intriguing insights into the mutual dependence of metabolism, SUCNR1 localization, and signaling.

## Materials and methods

### Chemicals, antibodies, commercial assays, and plasmids

All chemicals, antibodies, critical commercial assays, and plasmids used in this paper are listed in Table S1.

### Cell culture and transfection

The human embryonic kidney cell line (HEK293‐T, RRID:CVCL_0063) was acquired from the American Type Culture Collection (ATCC CRL‐3216, LGC Standards, Wesel, Germany). HEK293‐SUCNR1 and HEK293‐CTRL were kindly provided by Julien Hanson (University of Liège, Belgium) and are stably expressing a cAMP GloSensor22F with or without stable expression of human SUCNR1 [32]. All HEK293 cell lines were maintained in Dulbecco's Modified Eagle medium (DMEM) in a humidified 5% CO_2_ incubator at 37 °C and to all media 10% fetal bovine serum (FBS), 100 U·mL^−1^ penicillin and 100 μg·mL^−1^ streptomycin was added. Lipofectamine 2000 was used for transient transfection. Cells were split into 25 cm^2^‐cell culture flasks (HEK293‐T: 1.6 × 10^6^ cells/flask, HEK293 cells stably expressing SUCNR1: 2.5 × 10^6^ cells/flask). The following day, cells were transfected with plasmids (total amount of DNA: 4 μg). One day after transfection, cells were detached using Versene solution and seeded in poly‐l‐lysine coated plates (1 : 50 in PBS) to a density of 2.5 × 10^4^ cells (HEK293‐T) or 3 × 10^4^ cells (HEK293‐SUCNR1) in 96‐well‐plates or 1.2 × 10^5^ cells/well (HEK293‐T) in 48‐well‐plates for experiments.

The acute monocytic leukemia cell line THP‐1 (RRID:CVCL_0006) was acquired from the German Collection of Microorganisms and Cell Cultures (DSMZ, ACC 16). THP‐1 cells were maintained in Roswell Park Memorial Institute (RPMI) 1640 Medium in a humidified 5% CO_2_ incubator at 37 °C and to all media 10% heat‐inactivated FBS, 100 U·mL^−1^ penicillin and 100 μg·mL^−1^ streptomycin was added.

All experiments were performed with Mycoplasma‐free cells, as was regularly tested.

THP‐1 cells were differentiated according to the protocol published by Genin *et al*. [47]. In brief, THP‐1 cells were seeded in 96‐well plates (5 × 10^4^ cells/well), and phorbol‐12‐myristat‐13‐acetate (PMA) was added to a final concentration of 83 nm. Cells were differentiated 48 h later into pro‐inflammatory M1‐like macrophages using lipopolysaccharide (LPS, 20 ng·mL^−1^) and interferon γ (INFγ, 20 ng·mL^−1^) or anti‐inflammatory M2‐like macrophages using interleukin 4 (IL‐4, 20 ng·mL^−1^) and interleukin 13 (IL‐13, 20 ng·mL^−1^). THP‐1 cells were transfected with siRNA (2.5 pmol per well) and 0.5 μL TransIT‐TKO transfection reagent per well subsequent addition of M1‐ or M2‐polarizing medium. Assays were performed 48 h post‐transfection. For second messenger (cAMP, IP_1_) and ERK and Akt assays, THP‐1 cells were serum starved with 2.5% FBS in RPMI 16 h before the assay.

### DMR assay

The label‐free DMR assay was used to measure receptor activation. Transiently SUCNR1‐transfected HEK293‐T cells were seeded in fibronectin‐coated Epic 384‐well microplates (2.5 × 10^4^ cells/well) and cultured 24 h to reach a confluent monolayer. THP‐1 cells were seeded in Epic 384‐well microplates (5 × 10^4^ cells/well) and differentiated as described above. Equilibration was performed in PBS containing 0.4 mm MgCl_2_ and 0.4 mm CaCl_2_ supplemented with 10 mm glucose (PBS Glc) or 2 mm glutamine (PBS Gln), respectively, for 2 h, followed by stimulation with the respective agonists or the antagonist and DMR recording for 50 min. Cells were either untreated or treated with pertussis toxin (PTX, 100 ng·mL^−1^) 16 h or Ubo‐QIC (Ubo, 300 nm) 30 min before the assay.

### Cell‐surface ELISA

For cell surface expression analyses, HEK293‐T cells were seeded in T‐25 cell culture flasks as described above. Cells were co‐transfected the following day with HA‐tagged SUCNR1 (2 μg) and either dynamin‐2 wt, K44A or R399A (2 μg). ELISA was performed in 48‐well plates 48 h after transfection. Cell surface expression of N‐terminal HA‐tagged SUCNR1 was measured using an indirect cellular ELISA as described in Ref. [74].

### BRET assay

HEK293‐T cells were seeded in T‐25 cell culture flasks as described and transfected the following day. For experiments using PIP_2_ [36] (Fig. 8G) or determining receptor localization and miniG‐recruitment assays (Figs 7E–H, 10, and 11), cells were transfected with 2 μg receptor DNA and 2 μg of the respective co‐plasmid as indicated in the figure legend. For experiments using bi‐molecular biosensors and detecting active Gα_i_ (Fig. 12), HEK293‐SUCNR1 were seeded in T‐25 cell culture flasks and transfected 24 h later. Cells were transfected with donor : acceptor ratios of 1 : 10, that is, 0.4 μg Nluc‐tagged sensor KB1753 and 3.6 μg YFP‐tagged Gα_i_ protein. Experiments with bimolecular Gβγ‐sensors (Fig. 12) were transfected in HEK293‐SUCNR1 as follows: 1.5 μg Gα_i3_, 1 μg VC‐G_β1_, 1 μg VN‐G_γ2_, and 0.5 μg Nluc‐tagged sensor GRK3ct. Donor : acceptor ratios were re‐titrated for detection of active Gα_q_ and Gβγ in the presence of Gα_q_ (Fig. 12). The 2 × 10^5^ HEK293‐T cells/well were seeded and transfected with 150 ng of plasmid in total per well the following day. Reasonable results were obtained for active Gα_q_ using 75 ng SUCNR1 receptor plasmid, 74.0625 ng mVenus‐tagged Gα_q_, and 0.9375 ng Nluc‐tagged sensor GRK2RH per well, that is, donor:acceptor ratio of 1 : 40. Experiments with Gβγ‐sensors were transfected with the following amounts of plasmid per well: 50 ng SUCNR1 receptor plasmid, 50 ng Gα_q_, 16.45 ng VC‐G_β1_, 16.45 ng VN‐G_γ2_, and 0.625 ng Nluc‐tagged sensor GRK3ct, that is, donor : acceptor ratio of 1 : 80.

Control experiments with Y2R and M3R (Figs 7 and 10, 11, 12) were transfected in T25‐flasks with 1.06 μg receptor plasmid, 2.66 μg YFP‐tagged G protein, and 0.266 μg Nluc‐tagged sensor. Gβγ‐sensors were transfected in the following ratio: 1 μg receptor plasmid, 1.36 μg Gα_i3_ or Gα_q_, 0.545 μg VC‐G_β1_, 0.545 μg VN‐G_γ2_, and 0.545 μg Nluc‐tagged‐sensor.

Twenty‐four hours after transfection, cells were harvested and plated in white poly‐l‐lysine coated 96‐well cell culture plates. BRET assays were performed 48 h later. Assays with transfection in wells were performed 48 h after transfection. Therefore, cells were washed with PBS Glc or PBS Gln and equilibrated with 32.5 μL PBS Glc or PBS Gln in the absence or presence of NF56‐EJ40 for 60 min at room temperature. A 12.5 μL of NanoBRET NanoGlo substrate, diluted 1 : 250 (final dilution 1 : 1000) or 12.5 μL of 20 μm coelenterazine h (Rluc substrate, final 5 μm) in PBS Glc or PBS Gln were added, and three consecutive baseline reads were taken using the EnVision Microplate reader (Revvity, Lübeck, Germany). Luminescence was measured at 460 ± 80 nm (acceptor) and 645 ± 75 nm (donor) for Nluc or 460 ± 30 nm (acceptor), and 540 ± 30 nm (donor) for Rluc and BRET ratio was calculated as the ratio between acceptor and donor emission. Stimulation occurred by adding 5 μL of 10× ligand solution, and reading was continued for another 30 min. The basal BRET ratio before ligand stimulation was defined as the average of three consecutive BRET values. The agonist‐stimulated BRET ratio after ligand addition was defined as the average of three successive BRET values. To quantify ligand‐induced changes, ΔBRET was calculated for each well as % over basal [(Ratio_stim_ − Ratio_basal_)/Ratio_basal_] × 100. Subsequently, the average ΔBRET of vehicle control was subtracted.

### Plasmid construction

All primers used in this study are listed in Table S2. EGFP in the vector pEGFP‐N1 was replaced by mVenus, mRuby, or Nanoluciferase (Nluc) using the AgeI and NotI site (primers 1–5) to generate p‐mVenus‐N1, p‐mRuby‐N1, or p‐Nluc‐N1. HA‐SUCNR1‐FLAG in pcDps was generated as previously described (primers 6–11 [23, 75]). SUCNR1 was cloned in‐frame using the HindIII and the EcoRI site in p‐mVenus‐N1, p‐mRuby‐N1, or p‐Nluc‐N1 (primers 12–13).

Plasmids encoding FYVE‐KB1753‐Nluc (pcDNA3.1‐HA‐2xFYVE‐KB1753‐Nluc), FYVE‐GRK3ct‐Nluc (pcDNA3.1‐HA‐2xFYVE‐GRK3ct‐Nluc), and FYVE‐GRK2RH‐Nluc (pcDNA3.1‐HA‐2xFYVE‐GRK2RH‐Nluc) were generated by replacing the membrane anchoring sequence (mas) of the previously described plasmids pcDNA3.1‐mas‐KB1753‐Nluc, pcDNA3.1‐mas‐GRK3ct‐Nluc, and pcDNA3.1‐mas‐GRK2RH‐Nluc, respectively, by HA‐2xFYVE [42]. Briefly, pcDNA3.1‐mas‐KB‐1753‐Nluc, pcDNA3.1‐masGRK3ct‐Nluc, or pcDNA3.1‐mas‐GRK2RH‐Nluc were digested with NheI and HindIII to excise the mas cassette and subsequently ligate in the same sites a sequence corresponding to an HA‐tag followed by two copies of the FYVE domain of Hrs in tandem (Addgene, Watertown, MA, USA, p3E‐2xFYVE_hrs, Plasmid #67676). The 2xFYVE cassette included some silent mutations to facilitate cloning and was flanked on each end by sequences encoding two glycines.

Plasmids encoding KB1753‐Nluc‐rab7A, GRK3ct‐Nluc‐rab7A, and GRK2RH‐Nluc‐rab7A were generated by gene synthesis (Thermo Fisher Scientific, Darmstadt, Germany). The respective sequences KB1753, GRK3ct, and GRK2RH were in‐frame of Nluc and rab7A and inserted into pcDNA3.1 using EcoRI/HindIII or NheI/XhoI, respectively.

Quick change PCR was used to introduce mas in the mVenus‐mG_si43_ or mVenus‐mG_sq71_ construct [34], respectively (primers 14–15) and generate mas‐mVenus‐mG_si43_ and mas‐mVenus‐mG_sq71_. The constructs FYVE‐mVenus‐mG_si43_ and FYVE‐mVenus‐mG_sq71_ were generated by inserting HA‐2xFYVE in‐frame upstream of the respective miniG protein using NheI and XhoI as restriction sites (primers 16–20). Rab5A or Rab7A were inserted in frame downstream of mVenus‐miniG_si43_ and mVenus‐miniG_sq71_, respectively (primers 21–24 for rab7A, primers 25–27 for rab5A), to generate miniG proteins restricted to early or late endosomes.

The β‐arrestin‐2‐YFP construct (Addgene plasmid #36917 [76]) was used as a template (primers 28–29) to insert a NheI and a KpnI site upstream and downstream of the β‐arrestin‐2 open reading frame to insert it in p‐Nluc‐N1. The HA‐CHRM3‐FLAG in pcDps construct was used as a template (primers 30–31) to insert an EcoRI and a KpnI site upstream and downstream of the CHRM3 open reading frame to insert it in p‐Nluc‐N1, p‐mVenus‐N1, and p‐mRuby‐N1. Similarly, the HA‐Y2R‐FLAG in pcDps construct was used as a template (primers 32–33) to insert a NheI and an EcoRI site upstream and downstream of the Y2R open reading frame to insert it in p‐Nluc‐N1, p‐mVenus‐N1, and p‐mRuby‐N1.

The mouse Y2 receptor coding sequence was amplified from genomic mouse DNA, N‐terminally HA‐tagged and C‐terminally FLAG‐tagged (primers 34–37). The PCR product was digested with Aat II and Spe I and inserted into pcDps.

All PCR reactions were performed with Q5 High‐Fidelity DNA Polymerase following the manufacturer's instructions. All Quickchange PCR reactions were performed using Phusion High‐Fidelity DNA Polymerase. All primers were ordered from Seqlab. The correctness of all constructs was confirmed by sequencing (Seqlab, Göttingen, Germany).

### CQ1 confocal imaging

HEK293‐T cells were seeded into black 96‐well plates with a clear bottom (2 × 10^4^ cells/well) and transfected with 150 ng plasmid (single construct) or 75 ng of each construct (co‐transfection). The co‐localization of each receptor with the respective subcellular marker protein was calculated as the Pearson correlation coefficient (PCC) [77] using the JACoP plugin [78] in imagej (https://imagej.net/ij/). This plugin determines the relationship between the pixel intensities in two images, that is, receptor and subcellular marker.

Cells were fixed in 4% formaldehyde in PBS 24 h post‐transfection. Cells were stained with 2 μg·mL^−1^ Hoechst 33342 and stored in PBS. For images using CellMask plasma membrane stains (Invitrogen, Life Technologies, Thermo Fisher Scientific, Darmstadt, Germany), the medium was changed to PBS Glc containing 2 μg·mL^−1^ Hoechst 33342 and 5 μg·mL^−1^ CellMask plasma membrane stain and incubated for 30 min before imaging live.

For antibody staining, cells were incubated for 1 h in PBS Glc at 37 °C without CO_2_, fixed with 4% formaldehyde in PBS, and, if indicated in the figure, permeabilized with 0.5% Triton‐X in PBS. Cells were washed twice with PBS after each step. After that, medium with 10% FBS was added to each well, and cells were incubated for 30 min at 37 °C to block non‐specific binding sites. Cells were incubated with primary anti‐HA (1 : 1000), anti‐FLAG (1 : 500), anti‐clathrin (1 : 1000), anti‐caveolin (1 : 400), or anti‐EEA1‐antibody (1 : 100), respectively, for 2 h at 37 °C. After washing with PBS, the secondary antibody was added (1 : 500 diluted in medium), and cells were incubated for 1 h at 37 °C. For anti‐HA and anti‐FLAG, AlexaFluor555 goat anti‐mouse antibody was used. For anti‐clathrin, AlexaFluor488 goat anti‐mouse antibody was used. For anti‐caveolin and anti‐EEA‐1, AlexaFluor488 goat anti‐rabbit antibody was used. Subsequently, cells were washed with PBS, and nuclei were stained with 2 μg·mL^−1^ Hoechst for 10 min at 37 °C. Confocal images were taken with the CellVoyager CQ1 Benchtop High‐Content Analysis System.

THP‐1 cells polarized to M1‐ or M2‐like macrophages were transfected with red‐fluorescent siRNA (siTC). Cells were stained with 2 μg·mL^−1^ Hoechst 33342 and 5 μg·mL^−1^ CellMask plasma membrane stain and incubated for 30 min before imaging. Confocal images were taken with the CellVoyager CQ1 Benchtop High‐Content Analysis System.

### Alpha SureFire ultra multiplex pErk 1/2 and total Erk and pAkt1/2/3 and total Akt1 assay

The pErk/total Erk and pAkt/total Akt content of HEK293‐T and THP‐1 M2‐polarized cell extracts was determined by the Alpha SureFire Ultra Multiplex p‐ERK 1/2 (Thr202/Tyr204)/Total ERK assay and p‐Akt1/2/3 (Ser473) & Total Akt1 assay according to the manufacturer's protocol as previously described [79]. The kit measures the phosphorylation and total levels of endogenous ERK1/2 and AKT1/2/3 in cellular lysates. The signal at 615 nm (Eu) corresponds to the phosphorylated kinase level, while the signal at 545 nm (Tb) corresponds to the total kinase levels. HEK293‐T cells were split into 96‐well plates 24 h after transfection and serum‐starved 16 h before the assay. Stimulation with agonists was performed 48 h after transfection in PBS Glc or Gln for 10 min at 37 °C. When inhibitors were used, cells were pre‐incubated with the inhibitor in respective buffer (Glc or Gln) at 37 °C for 30 min before agonist stimulation or 16 h prior to the assay (pertussis toxin, PTX, 100 ng·mL^−1^). The 2‐fold concentrated agonist was added to inhibitor‐containing wells to prevent wash‐out effects. Reactions were stopped by media aspiration, and cells were lysed in 50 μL of supplied lysis buffer supplemented with 250 μm protease inhibitor AEBSF. From each well, 10 μL of lysate was transferred to a 384‐well plate. Acceptor beads and donor beads were added according to the manufacturer's protocol. Measurements were taken using the EnVision Microplate reader.

### ALPHAScreen cAMP assay

The cAMP content of cell extracts was determined by a non‐radioactive assay based on the ALPHAScreen technology according to the manufacturer's protocol as previously described [75].

### Cisbio HTRF inositol monophosphate (IP_1_) assay

IP_1_ content of cell lysates was determined using the Cisbio IP‐One Gq kit according to the manufacturer's protocol. Cells were split into 96‐well plates 24 h after transfection. Stimulation with agonists was performed 48 h after transfection in 35 μL stimulation buffer for 30 min at 37 °C. Cells were lysed by direct addition of 30 μL ice‐cold lysis buffer. Acceptor and donor beads were added according to the manufacturer's protocol. Measurements were taken using the EnVision Microplate reader and calculated as acceptor emission (665 nm) over donor emission (620 nm).

### Seahorse XF cell mito stress test

Metabolic analyses were performed using the Seahorse XF Cell Mito Stress Test Kit and the XFe96 Analyzer (Agilent Technologies, Waldbronn, Germany) as described previously [23]. HEK293‐SUCNR1 and HEK293‐CTRL cells were seeded in poly‐l‐lysine coated XF96 cell culture microplates (HEK293‐SUCNR1: 3 × 10^4^ cells/well; HEK293‐CTRL: 1.5 × 10^4^ cells/well) and incubated at 37 °C and 5% CO_2_ for 48 h. THP‐1 cells were seeded (5 × 10^4^ cells/well) and differentiated with a siRNA‐mediated knockdown, as described above. Pertussis toxin (PTX) was added 16 h before the assay to a final concentration of 100 ng·mL^−1^. On the day of the assay, cells were washed twice with XF RPMI medium (37 °C; pH 7.4) supplemented with either Glc (10 mm) or Gln (2 mm). Finally, 180 μL of the assay medium in the absence or presence of agonists or Ubo (final 300 nm) was added to the cells, and the microplates were placed in a 37 °C incubator without CO_2_ for 1 h. The respiratory chain inhibitors Oligomycin A, Carbonyl cyanide‐4 (trifluoromethoxy) phenylhydrazone (FCCP), and Rotenone/Antimycin A that are used for the determination of different mitochondrial key parameters were solved and loaded into the ports of the sensor cartridge following the manufacturer's protocol. The electron transport chain (ETC) consists of complex I (inhibited by Rotenone), complex II/SUC dehydrogenase, complex III (inhibited by Antimycin A), and complex IV. The electrochemical proton gradient generated by the electron transport is used for ATP generation by complex V/ATP synthase (inhibited by Oligomycin A). FCCP is a chemical uncoupler that disrupts the proton gradient and mitochondrial membrane potential, allowing for maximal oxygen consumption. The final well concentrations in the experiments were 1.5 μm Oligomycin A, 2 μm FCCP, and 0.5 μm Rotenone/Antimycin A. After 15–30 min calibration of the cartridge in the XFe96 Analyzer, the utility plate was replaced with the cell culture microplate, and the assay was started. OCR and ECAR were determined every 6 min for 18 min under basal conditions as well as after consecutive Oligomycin A, FCCP, and Rotenone/Antimycin A injections. After the assay, cells were stained with Hoechst (1 μg·mL^−1^), and the cell number in each well was determined with the Celigo Image Cytometer (Revvity, Lübeck, Germany). Finally, OCR and ECAR were normalized to 10^4^ cells.

### Calcium imaging

HEK293‐T cells were transfected with mRuby‐tagged SUCNR1. Transfected cells (2 × 10^5^ cells/well) were seeded into 24‐well plates on poly‐l‐lysine coated glass coverslips, and calcium imaging was carried out 24–48 h post‐transfection as previously described [79]. Cells were loaded with 5 μm fura‐2‐AM in a standard solution containing 140 mm NaCl, 10 mm HEPES, 5 mm KCl, 2 mm CaCl_2_, 1 mm MgCl_2_, and 10 mm glucose for at least 60 min. For measurement of the glutamine‐ or BPTES‐dependent SUCNR1 response, cells were loaded with fura‐2‐AM in a standard solution containing 2 mm glutamine with or without 10 μm BPTES instead of glucose, respectively, for at least 60 min [80]. Fura‐2‐AM‐based calcium imaging was performed in single cells using a monochromator‐based imaging system and the imaging software tillvision 4.0 (T.I.L.L. Photonics, Gräfelfing, Gerany). Emitted fluorescence (excited at 340 and 380 nm) was acquired with a CCD camera (PCO Imaging) at intervals of 2 s and corrected for background fluorescence. Transfected cells were detected by emitted mRuby fluorescence, excited at 550 nm. Agonists of SUCNR1, as well as ATP, were dissolved in a standard solution and applied to the cells by bath perfusion.

### RNA preparation, reverse transcription, and real‐time quantitative PCR

SUCNR1‐ or empty vector‐transfected HEK293‐T cells were seeded into poly‐l‐lysine coated 12‐well plates (2.5 × 10^5^ cells/well). The cells were incubated in PBS Glc or Gln for 4 h at 37 °C the following day before harvesting in 500 μL BL + TG buffer per well. mRNA was isolated using the ReliaPrep RNA Cell Miniprep System following the company's protocol as previously described [23], and yielded RNA was stored at −80 °C. The RNA concentration was determined with the NanoDrop ND‐1000, and an additional DNase I digestion was performed at 37 °C for 30 min before reverse transcription to remove any remaining DNA. Afterward, DNase I was inactivated by adding EDTA (5 mm) and incubating at 75 °C for 10 min. Finally, the iScript cDNA Synthesis Kit was used to transcribe RNA in cDNA. Real‐time quantitative PCR (qPCR) was performed with 1 μL cDNA, 1 μL primer mix with sense‐ and antisense‐primer (400 nm of each primer), 5 μL Luna Universal qPCR Master Mix, and 5 μL nuclease‐free water on the CFX Connect Real‐Time PCR Detection System. qPCR was initiated with the activation of the DNA polymerase at 95 °C for 2 min followed by 40 cycles of denaturation at 95 °C for 15 s and primer annealing and elongation at 60 °C for 30 s. Fluorescence was measured at the end of each annealing/elongation step. Melting curves were recorded from 55 °C to 95 °C (0.5 °C increment, 5 s per step) and showed one single peak for each primer pair. Primers were designed with primer‐blast (http://www.ncbi.nlm.nih.gov/tools/primer‐blast/) and purchased from Microsynth Seqlab (primers 38–69, Table S2). For the calculation of Δ*C*_t_ values, the mRNA expression of the reference genes ACTB and RPS18 was determined.

### Synthesis of (6‐(4‐(trifluoromethoxy)phenyl)picolinoyl)‐l‐aspartic acid (**31**)

All starting materials and reagents were of commercial quality and were used without further purification. The compound **31** (Fig. 4, Fig. S1) was synthesized in two steps [27]. First, dimethyl (6‐(4‐(trifluoromethoxy))phenyl)picolinoyl)‐l‐aspartate (**C**) was synthesized by the amide coupling reaction (Fig. S1). To the solution of compound (6‐(4‐trifluoromethoxyphenyl)picolinic acid (**A**, 283 mg, 10 mmol), dimethyl l‐aspartate‐hydrochloride (**B**, 197 mg, 10 mmol), and O‐(7‐azabenzotriazol‐1‐yl)‐*N*,*N*,*N*′,*N*′‐tetramethyluronium hexafluorophosphate (HATU) (480 mg, 11 mmol) in dimethylformamide (10 mL) at room temperature, diisopropylethylamine (0.45 mL, 25 mmol) was added (Fig. S1). The mixture was stirred overnight. After completion of the reaction (monitored by thin layer chromatography [TLC]), the mixture was poured into water and extracted with ethyl acetate (2 × 25 mL). The combined organic layers were washed with a brine solution (25 mL), dried over Na_2_SO_4_, and evaporated under reduced pressure to dryness. Purification by flash column chromatography gave the ester intermediate **C** as a sticky oil (Fig. S1). Yield 90% (358 mg). ^1^H NMR (400 MHz, DMSO‐*d*_*6*_) δ = 9.20–9.15 (m, 1H), 8.40–8.36 (m, 2H), 8.27–8.22 (m, 1H), 8.15–8.11 (m, 1H), 8.05–8.02 (m, 1H), 7.53–7.43 (m, 1H), 5.10–5.06 (m, 1H), 3.81 (s, 3H), 3.75 (s, 3H), 3.06–3.02 (m, 2H). ESI‐HRMS calcd for C_19_H_17_F_3_N_2_NaO_6_ [M + Na]^+^ 449.0931, found 449.0937.

The ester **C** (213 mg, 5 mmol) was subsequently hydrolyzed to **31** in the presence of 2 N sodium hydroxide (1 mL) at room temperature overnight in methanol (5 mL). After completion of the reaction (monitored by TLC), the solution was evaporated under reduced pressure to dryness. The residue was dissolved in water and cooled to ice‐water temperatures. The 2 N hydrochloric acid (10 mL) was slowly added to the solution. The resulting precipitation was filtered, washed with water (20 mL), and dried under reduced pressure. White solid Yield 98% (195 mg) ^1^H NMR (400 MHz, DMSO‐*d*_*6*_) δ = 12.62, (br s, 2H), 9.24 (d, *J* = 8.8 Hz, 1H), 8.41–8.38 (m, 2H), 8.26 (dd, *J* = 7.9, 0.8 Hz, 1H), 8.14 (t, *J* = 7.8 Hz, 1H), 8.05 (dd, *J* = 7.6, 0.8 Hz, 1H), 7.53 (d, *J* = 8.1 Hz, 1H), 4.89–4.85 (m, 1H), 2.91 (dd, *J* = 11.1, 4.7 Hz, 2H). ^13^C NMR (101 MHz, DMSO‐*d*_*6*_) δ = 172.76 (d, *J* = 13.8 Hz), 163.77, 154.06, 149.76 (d, *J* = 14.4 Hz), 139.73, 137.16, 129.40, 123.79, 121.74, 121.49. ESI‐HRMS calcd for C_17_H_12_F_3_N_2_O_6_ [M − H]^−^ 397.0653, found 397.0659. HPLC *t*_R_ = 7.26 (100% purity).

As previously described [81], TLC was carried out on Merck 60 F254 silica plates (Merck KGaA, Darmstadt, Germany), and spots were visualized under UV light (254 and 366 nm) or developed with an appropriate staining reagent. Flash chromatography was carried out on an Interchim PuriFlash XS420flash chromatography system and Grace Davison Davisil LC60A 20e45 mm silica or Merck Geduran Si60 63–200 μm.

Nuclear magnetic resonance (NMR) spectra were recorded on Bruker Avance 400 instruments (Bruker Corporation, Billerica, MA, USA) as previously described [81, 82]. The samples were dissolved in deuterated solvents, and chemical shifts are given in parts per million in relation to tetramethylsilane (TMS). Spectra were calibrated using the solvent's residual proton or carbon peaks. Mass spectrometry (MS) was carried out with an Advion TLC–MS interface (Advion, Ithaca, NY, USA) with electrospray ionization (ESI) in positive and/or negative mode. Instrument settings were as follows: ESI voltage of 3.50 kV, capillary voltage of 187 V, source voltage of 44 V, capillary temperature of 250 °C, desolvation gas temperature of 250 °C, and gas flow of 5 L of nitrogen per minute.

As previously described [82], the purity of the final compound was determined via high‐performance liquid chromatography (HPLC) using an Agilent 1100Series LC system (Agilent Technologies, Santa Clara, CA, USA) with a Phenomenex Kinetex C8 100A column (150 mm × 4.6 mm, 2.6 μm) (Phenomenex Inc., Torrance, CA, USA), and detection was performed with a UV DAD at wavelengths of 254 and 230 nm. Elution was carried out with the following gradients: 0.01 m KH_2_PO_4_ (pH 2.32) (solvent A) and MeOH (solvent B). Method A: 0 min, 40% B/6 0% A; 9 min, 95% B/5% A; 10 min, 95% B/5% A; 11 min, 40% B/60% A; 16 min, 40% B/60% A; flow of 0.5 mL·min^−1^. Method B: 0 min, 40% B/60% A; 15 min, 85% B/15% A; 20 min, 85% B/15% A; 22 min, 40% B/60% A; 28 min, 40% B/60% A; flow 0.5 mL·min^−1^. The final compounds showed a purity of > 95% according to the peak areas at the two different wavelengths.

### Gas chromatography–mass spectrometry (GC–MS) analyses of SUC in cells and medium

One day post‐transfection, transiently with SUCNR1 or empty vector‐transfected HEK293‐T cells were seeded in poly‐l‐lysine‐coated (1 : 50 in PBS) 6‐well plates (1 × 10^6^ cells/well) and incubated for 24 h at 37 °C and 5% CO_2_. Cells were washed with PBS Glc or PBS Gln and incubated with the respective medium for 2 h at 37 °C. The plates were placed on ice, the medium was removed from the plates, and 0.5 mL MeOH : H_2_O (70 : 30) was added to each well. Cells were harvested using a plastic cell scraper and transferred to pre‐chilled tubes. The lysate was extracted for 5 min using ultrasonication and centrifuged for 10 min at 4 °C with 24 000 ***g***. A 400 μL of the lysate was transferred to a fresh tube, and samples (medium and cell lysate) were dried in a vacuum concentrator and stored at −20 °C until detection.

Analysis of SUC was carried out according to Hutschenreuther *et al*. [83]. Briefly, vacuum‐dried extracts (*n* = 6) were incubated by shaking in methoxyamine hydrochloride in pyridine and *N*,*O*‐bis(trimethylsilyl)‐trifluoroacetamide. After derivatization, samples were transferred to glass vial micro‐inserts and subjected to GC–MS analysis on an Agilent 6890 gas chromatograph coupled to an Agilent 5973N quadrupole mass selective detector (Agilent Technologies, Böblingen, Germany) with standard electron impact ionization (70 eV). Separation was accomplished on a DB‐5MS Ultra Inert column (Agilent, Waldbronn, Germany; 30 m × 0.25 mm ID and 0.25 μm film) at 1.2 mL·min^−1^ carrier gas flow (He 5.0 Alphagaz; Air Liquide, Leipzig, Germany) after splitless injection of 1 μL sample at 280 °C as previously described [84]. The oven program started at 40 °C with a 6 °C·min^−1^ temperature increase up to 325 °C and a final time of 14 min. Compound identification was based on co‐spiking of authentic standards of SUC. Quantitation was accomplished in a standard addition‐like approach using sample aliquots for spiking different calibration levels (12 levels, *n* = 3). Mass Hunter Quantitative Analysis B.07.00 (Agilent Technologies, Böblingen, Germany) was used to establish a method for automated peak finding and integration of signals corresponding to SUC. All calculations were performed in MS Excel 2016.

### Data analyses

All data were statistically analyzed and visualized using graphpad prism 7 (GraphPad Software, Boston, MA, USA) for Windows. Detailed information about statistical analyses is included in each figure legend.

## Conflict of interest

The authors declare no conflict of interest.

## Author contributions

A‐DL and CS contributed to conceptualization, writing—original draft, visualization, and funding acquisition; A‐DL, AS, FB, SB, CB, TP, MG‐M, RK, and CS contributed to methodology; A‐DL, PK, RK, and CS contributed to validation; A‐DL, PR, CZ, FB, CB, TP, RK, and CS contributed to formal analysis; A‐DL, PR, PK, CZ, FB, SB, CB, TP, RK, and CS contributed to investigation; AS, CB, TP, MG‐M, RK, and CS contributed to resources; A‐DL, PR, CZ, CB, TP, and CS contributed to data curation; A‐DL, RK, and CS contributed to writing—review and editing; CS contributed to supervision and project administration. All authors discussed the results and implications and commented on the manuscript at all stages. All authors read and approved the final manuscript.

### Peer review

The peer review history for this article is available at https://www.webofscience.com/api/gateway/wos/peer‐review/10.1111/febs.17407.

## Supporting information


**Fig. S1.** Reagent and conditions to synthesize compound **31**.
**Table S1.** Materials.
**Table S2.** Primers used for quantitative RT‐PCR (qPCR) and generation of fluorescent receptors, miniG variants, and active G protein sensors.

## Data Availability

All data that supports the findings of this study are available in this article and the Supporting Information of this article.
