# Supplementary material for: Succinate receptor 1 signaling mutually depends on subcellular localization and cellular metabolism
Source: FEBS J. 2025 Jan 21;292(8):2017–50. doi: 10.1111/febs.17407 (PMC12001207; doi:10.1111/febs.17407)
Supplement: Supplementary file 1 — Fig. S1. Reagent and conditions to synthesize compound 31. Table S1. Materials. Table S2. Primers used for quantitative RT‐PCR (qPCR) and generation of fluorescent receptors, miniG variants, and active G protein sensors. [file FEBS-292-2017-s001.pdf]

Supplemental information

Succinate receptor 1 signaling mutually depends on subcellular localization and cellular metabolism

Aenne-Dorothea Liebing[1], Philipp Rabe[1], Petra Krumbholz[1], Christian Zieschang[1], Franziska Bischof[1], Angela Schulz[1], Susan Billig[2], Claudia Birkemeyer[2,3], Thanigaimalai Pillaiyar[4], Mikel Garcia-Marcos[5,6], Robert Kraft[7], Claudia Stäubert[1]*

[1] Rudolf Schönheimer Institute of Biochemistry, Medical Faculty, Leipzig University, Johannisallee 30, 04103, Leipzig, Germany

[2] Research Group of Mass Spectrometry, Institute of Analytical Chemistry, Leipzig University, Linnéstraße 3, 04103, Leipzig, Germany

[3] German Center for Integrative Biodiversity Research (iDiv) Halle-Leipzig-Jena, 04103 Leipzig, Germany

[4] Institute of Pharmacy, Pharmaceutical/Medicinal Chemistry and Tübingen Center for Academic Drug Discovery, Eberhard Karls University Tübingen, Tübingen, Germany

[5] Department of Biochemistry & Cell Biology, Chobanian & Avedisian School of Medicine, Boston University, Boston, Massachusetts, USA

[6] Department of Biology, Boston University College of Arts & Sciences, Boston, Massachusetts, USA

[7] Carl Ludwig Institute for Physiology, Medical Faculty, Leipzig University, Leipzig, Germany

Running title: Metabolic regulation of SUCNR1 signaling

*To whom correspondence and requests for materials should be addressed:

Claudia Stäubert, Rudolf Schönheimer Institute of Biochemistry, Faculty of Medicine, Leipzig University, Johannisallee 30, 04103 Leipzig, Germany, Tel.: +49-341-9722-157, Fax: +49-341-9722-159, Email: claudia.staeubert@medizin.uni-leipzig.de

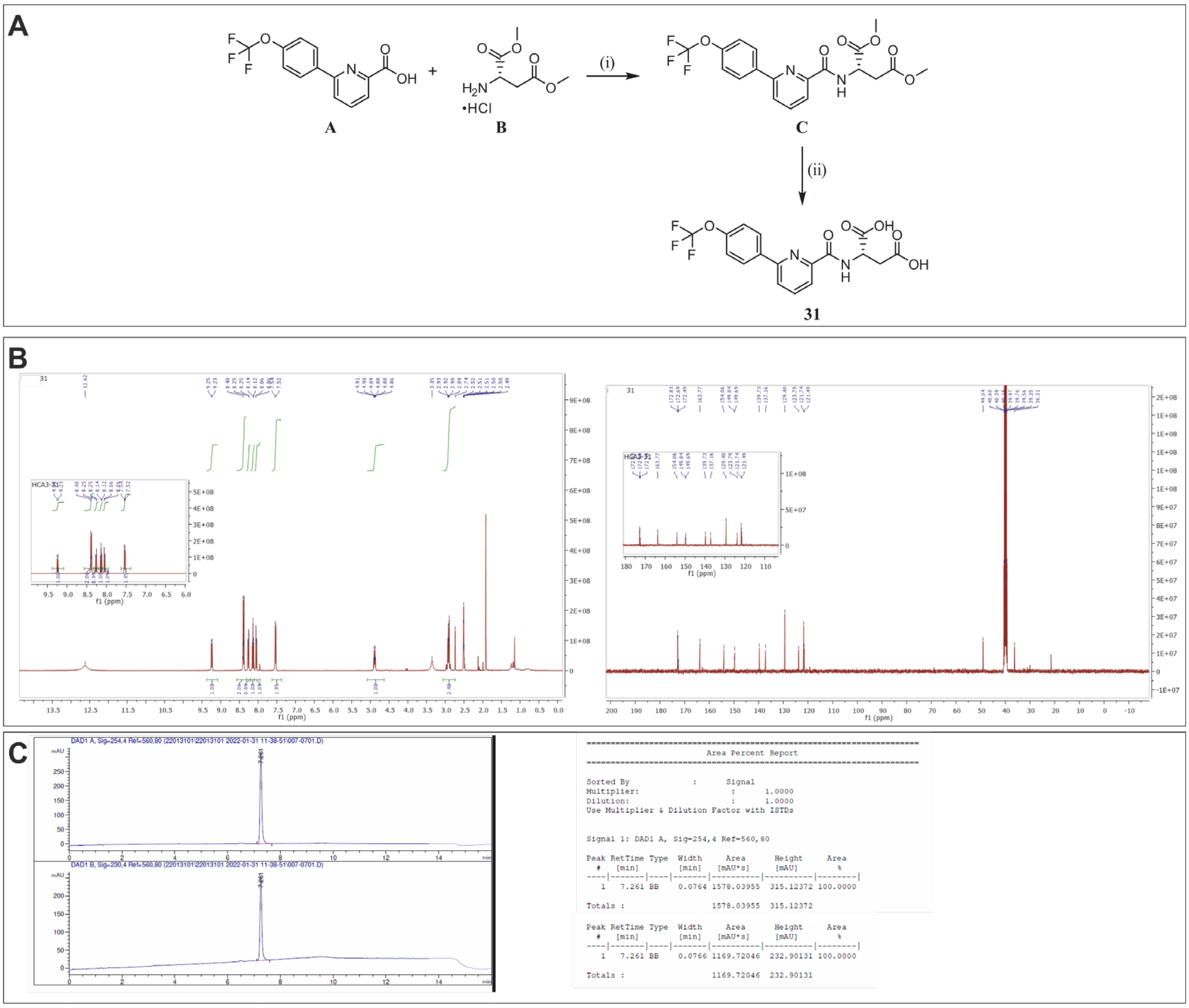

Supplementary Figure S1: Reagent and conditions to synthesize compound 31 (i) HATU, DIPEA, DMF, 12 h, rt. 90 %; (ii) 2 N NaOH, methanol, 12 h, rt, 98%. **(B)** ^{1}H- and ^{13}-C NMR spectra of 31 (C) HPLC purity data of **31.** Related to Methods.

Supplementary Table S1: Materials.

**Antibodies**		
Anti-HA-Peroxidase, High Affinity from rat IgG1 monoclonal	Sigma-Aldrich	12013819001RRID: AB_390917
Anti-HA antibody produced in mouse	Sigma-Aldrich	H3663
Anti-FLAG M2 antibody produced in mouse	Sigma-Aldrich	F1804
Goat anti-Mouse IgG (H+L) Secondary Antibody, Alexa Fluor 555	Thermo Fisher Scientific	A21422
Goat anti-Mouse IgG (H+L) Secondary Antibody, Alexa Fluor 488	Thermo Fisher Scientific	A11029
Goat Anti-Rabbit IgG (H+L) F(ab')2 Fragment Secondary Antibody, Alexa Fluor 488	Cell Signaling Technologies	4412
Mouse Clathrin Heavy Chain Monoclonal Antibody (X22)	Thermo Fisher Scientific	MA1-065
Rabbit Caveolin-1 (D46G3) XP Monoclonal Antibody	Cell Signaling Technologies	3267
Rabbit EEA1 (C45B10) Monoclonal antibody	Cell Signaling Technologies	3288
**Chemicals, peptides, and recombinant proteins**		
10x HBSS	Thermo Fisher Scientific	14065056
1x D-PBS	Thermo Fisher Scientific	14190144
6-(4-trifluoromethoxyphenyl)picolinic acid	Abcr	AB325212
Antimycin A	Santa Cruz	sc-202467
Barbadin	MedChemExpress	HY-119706
Bis-2-(5-phenylacetamido-1,3,4-thiadiazol-2-yl)ethylsulfid (BPTES)	Sigma-Aldrich	SML0601
Carbachol	Sigma-Aldrich	Y0000113
Carbonyl cyanide-p-trifluoromethoxyphenylhydrazone (FCCP)	Santa Cruz	sc- 203578
CellMask Plasma Membrane Stain green	Thermo Fisher Scientific	C37608
cis-epoxysuccinic acid (CES)	TCI chemicals	E0449
Citric acid	Sigma-Aldrich	251275
Coelenterazine h	Biomol	ABD-21159
Deoxynucleotide (dNTP) Solution Set	NEB	N0446
Diisopropylethylamine	Sigma-Aldrich	D125806
Dimethyl sulfoxide	Sigma-Aldrich	D8418
DNase I	NEB	M0303S
Dulbecco's Modified Eagle Medium (DMEM)	Thermo Fisher Scientific	41966029
Fetal bovine serum (FBS)	Thermo Fisher Scientific	10270106
Formaldehyde	Sigma-Aldrich	F8775

Forskolin	Sigma-Aldrich	F3917
Fura 2-AM	Sigma-Aldrich	47989
Gallein	MedChemExpress	HY-D0254
H_2O_2	Sigma-Aldrich	1.08600
HCl	Sigma-Aldrich	258148
HEPES (1M)	Thermo Fisher Scientific	15630056
Hoechst 33342	Thermo Fisher Scientific	H1399
Interferon γ	Sigma-Aldrich	SRP3058
Interleukin 4 (IL-4) human	Sigma-Aldrich	H7291
Interleukin 13 (IL-13) human	Sigma-Aldrich	SRP3076
Itaconate	Sigma-Aldrich	I29204
L-Aspartic acid dimethyl ester hydrochloride	Abcr	AB136782
Lipofectamine 2000	Thermo Fisher Scientific	11668019
Lipopolysaccharide from *E. coli*	Sigma-Aldrich	L4516
L-Malic acid	Sigma-Aldrich	M9138
Malonic acid	Sigma-Aldrich	M1296
Methoxyamine hydrochloride	Alfa Aesar by Thermo Scientific	11388145
Methylmalonic acid	Sigma-Aldrich	M54058
N,N-Dimethylformamide	Sigma-Aldrich	494488
N,O-bis(trimethylsilyl)-trifluoroacetamide	Macherey-Nagel	REF 701220.110
$Na_2HPO_4 \cdot 2\ H_2O$	Sigma-Aldrich	S9763
Na_2SO_3	Sigma-Aldrich	S0505
NF56-EJ40	Axon Medchem	3056
NPY peptide	Core Unit Peptide Technologies Leipzig University	N/A
O-(7-azabenzotriazol-1-yl)-*N,N, N′,N′*-tetramethyluronium hexafluorophosphate	Abcr	AB545523
Oligomycin A	Santa Cruz	sc-201551
o-phenylenediamine	Sigma-Aldrich	P23938
Opti-MEM	Thermo Fisher Scientific	31985070
Oxaloacetic acid	Sigma-Aldrich	O9504
Pefabloc SC (AEBSF)	Sigma-Aldrich	11429868001
Penicillin-Streptomycin (5.000 U/ml)	Thermo Fisher Scientific	15140122
Pertussis toxin (PTX)	Sigma-Aldrich	P7208-50UG
Phorbol-12-myristat-13-acetate (PMA)	Sigma-Aldrich	P1585-1MG

Phusion High-Fidelity Polymerase	Thermo Fisher Scientific	F530S
Poly-L-lysine solution (0.1% (w/v) in H_2O)	Sigma-Aldrich	P8920
Pyridine	Sigma-Aldrich	270970
Q5 High-Fidelity DNA Polymerase	NEB	M0491
Roswell Park Memorial Institute (RPMI) 1640 Medium	Thermo Fisher Scientific	21875034
Rotenone	Santa Cruz	sc-203242
Sodium citrate	Sigma-Aldrich	S4641
Sodium hydroxide	Sigma-Aldrich	655104
Sodium sulfate	Sigma-Aldrich	S6547
Succinate	Sigma-Aldrich	398055
TransIT-TKO	Biozol	MIR-2150
Triton X-100	Sigma-Aldrich	T8787
Trypsin-EDTA (0.25%), phenol red	Thermo Fisher Scientific	25200056
Ubo-QIC (FR900359)	Biomol	Cay33666
UltraPure 0.5 M EDTA, pH 8	Thermo Fisher Scientific	15575020
Versene	Thermo Fisher Scientific	15040033
**Critical commercial assays**		
AlphaScreen cAMP kit	PerkinElmer Life Sciences	6760635M
Alpha SureFire Ultra Multiplex Phospho/Total ERK1/2 Assay Kit	PerkinElmer Life Sciences	MPSU-PTERK-M10K
Alpha SureFire Multiplex Surefire Ultra Phospho-AKT1/2/3 (Ser473 & AKT1 Total) Detection Kit	PerkinElmer Life Sciences	MPSU-PTAKT-M10K
IP-One Gq kit	Cisbio	62IPAPEC
iScript cDNA synthesis kit	BioRad	1708891
Luna Universal qPCR Master Mix	NEB	M3003E
NanoBRET NanoGlo substrate	Promega	N1572
**siRNA**		
siSUCNR1-I rArCrUrGrGrUrUrUrCrUrUrCrCrUrCrUrArArGrArAr UrUGA	Origene	SR324591B
siSUCNR1-II rCrArUrGrGrArArUrGrCrArArCrUrUrGrCrArArArAr ArCTG	Origene	SR324591C
Non coding siRNA (siNC) rCrGrUrUrArArUrCrGrCrGrUrArUrArArUrArCrGrCr GrUAT	Origene	SR30004
TYE 563 fluorescent-labeled transfection control siRNA duplex (siTC)	Origene	SR30002

**Recombinant DNA**		
p-mRuby-N1	pEGFP-N1	This paper
p-mVenus-N1	pEGFP-N1	This paper
p-Nluc-N1	pEGFP-N1	This paper
HA-human-SUCNR1-FLAG	pcDps	[1]
human-SUCNR1-mRuby	pEGFP-N1	This paper
human-SUCNR1-mVenus	pEGFP-N1	This paper
human-SUCNR1-Nluc	pEGFP-N1	This paper
rat-dynamin-2 wt	pcDps	[2]
rat-dynamin-2 K44A	pcDps	[2]
rat-dynamin-2 R399A	pcDps	[2]
mVenus-rab5A	pEGFP-N1	[3]
mVenus-rab7A	pEGFP-N1	[3]
mVenus-rab11A	pEGFP-N1	[3]
mas-KB1753-Nluc	pcDNA3.1	[4]
mas-GRK2RH-Nluc	pcDNA3.1	[4]
mas-GRK3ct-Nluc	pcDNA3.1	[5]
FYVE-KB1753-Nluc	pcDNA3.1	This paper
FYVE-GRK2RH-Nluc	pcDNA3.1	This paper
FYVE-GRK3ct-Nluc	pcDNA3.1	This paper
KB1753-Nluc-rab7A	pcDNA3.1	This paper
GRK2RH-Nluc-rab7A	pcDNA3.1	This paper
GRK3ct-Nluc-rab7A	pcDNA3.1	This paper
mVenus-mG$_{si43}$	pEGFP-N1	[6]
mVenus-mG$_{sq71}$	pEGFP-N1	[6]
mas-mVenus-mG$_{si43}$	pEGFP-N1	This paper
FYVE-mVenus-mG$_{si43}$	pEGFP-N1	This paper
mVenus-mG$_{si43}$-rab5A	pEGFP-N1	This paper
mVenus-mG$_{si43}$-rab7A	pEGFP-N1	This paper
mas-mVenus-mG$_{sq71}$	pEGFP-N1	This paper
FYVE-mVenus-mG$_{sq71}$	pEGFP-N1	This paper
mVenus-mG$_{sq71}$-rab5A	pEGFP-N1	This paper
mVenus-mG$_{sq71}$-rab7A	pEGFP-N1	This paper
Gα_{i3}-YFP	pcDNA3.1	[7]
Gα_{i3} in pcDNA3.1	pcDNA3.1	[8]

$G\alpha_q$-mVenus	pcDNA3.1	[9]
$G\alpha_q$ in pcDNA3.1	pcDNA3.1	[10]
pcDNA3.1-$G\beta_1$	pcDNA3.1	[11]
pcDNA3.1-$G\gamma_2$	pcDNA3.1	[11]
mVenus (155-239) $Gß_1$	pcDNA3.1	[11]
mVenus (1-155) Gy_2	pcDNA3.1	[11]
Nluc-β-arrestin-2	pEGFP-N1	This paper
PM-Venus-T2A-PLCδ-PH-Luc (PIP2)	bicistronic T2A vector	[12]
rat-CHRM3	pcDps	[13]
rat-CHRM3-mVenus	pEGFP-N1	This paper
rat-CHRM3-Nluc	pEGFP-N1	This paper
rat-CHRM3-mRuby	pEGFP-N1	This paper
mouse-Y2R	pcDps	This paper
mouse-Y2R-mVenus	pEGFP-N1	This paper
mouse-Y2R -Nluc	pEGFP-N1	This paper
mouse-Y2R-mRuby	pEGFP-N1	This paper
pcDps	[14]	N/A
pcDNA3.1	Invitrogen	N/A
EGFP-N1	Clontech	N/A
**Software and algorithms**		
GraphPad Prism 7	Graphpad Software	https://www.graphpad.com/scientific-software/prism/
Biorender	Biorender	https://biorender.com/
DNASTAR Lasergene	DNASTAR Lasergene	https://www.dnastar.com/software/lasergene/
CorelDraw	Corel	https://www.coreldraw.com/
SnapGene Viewer	SnapGene	https://www.snapgene.com/snapgene-viewer
Microsoft Excel 2016	Microsoft	https://www.microsoft.com/de-de/microsoft-365/excel
Image J	National Institutes of Health	https://imagej.nih.gov/ij/
CFX manager	BioRad	https://www.bio-rad.com/
**Other**		
384-well cyclic olefin copolymer EPIC biosensor plates	Corning	5040

384-well fibronectin-coated EPIC biosensor plates	Corning	5042
Celigo Image Cytometer	Nexcelom Bioscience LLC./ Cenibra GmbH	200-BFFL-S
CellVoyager CQ1 Benchtop High-Content Analysis System	Yokogawa	N/A
EnVision 2104 multilabel plate reader	PerkinElmer Life Sciences	2104-9030
Fresco 21 microcentrifuge	Thermo Fisher Scientific	75002555
Genevac miVac Duo Centrifugal Concentrator	Thermo Fisher Scientific	10098362
Seahorse XFe96 analyzer	Agilent Technologies	101991-100
Seahorse XFe96 FluxPak	Agilent Technologies	103792-100
TILLvisION 4.0	T.I.L.L. Photonics	N/A
Bio-Rad CFX Connect 96-Well qPCR Real-Time Cycler	BioRad	7531

Supplementary Table S2: Primers used for quantitative RT-PCR (qPCR) and generation of fluorescent receptors, miniG variants, and active G protein sensors.

no	primer name	primer sequence (5' – 3')
1	AgeI-Kozak-Venus-S	atACCGGTCGCAgtgagcaagggcgag
2	AgeI-Kozak-Ruby-S	atACCGGTCGCAgtgtctaagggcgaaga
3	AgeI-Kozak-NLuc-S	atACCGGTCGCAgtcttcacactcgaagatt
4	NotI-Stop-VenusRuby-AS	GATgcggccgcTTActtgtacagctcgtc
5	NotI-Stop-NLuc-AS	GATgcggccgcTTAcgccagaatgcgt
6	human SUCNR1-5UTR-S	ggcagagttcctgtcaaggg
7	human SUCNR1-3UTR-AS	cttgtcgtcccattcttttcaca
8	human SUCNR1-HA-adaptor	acgtccccgactacgccCTGGGGATCATGGCATGGAA
9	human SUCNR1-FLAG-adaptor	tcacttatcgtcatcgtccttatagtcCTTTTCTCTGAATGAAAGTAGGAG TTC
10	HA-universal primer EcoRI-S	cgcGAATTCgccaccATGtacccctacgacgtccccgactacg
11	FLAG-universal primer SpeI-AS	cgccgcACTAGTTCActtatcgtcatcgtccttatagtc
12	HindIII-Kozak-ATG-SUCNR1-S	TTCaagcttGCCACCatgctggggatcatgg
13	EcoRI-no-Stop-SUCNR1-AS	TGCAGAATTCgcttttctctgaatgaaagtagg
14	mas-linker-QC-miniG-S	GTCGCCACCATGGGGAGTAGCAAGAGCAAGGGAATCAA GCTTGGTGGAGTGAGCAAGGGCG
15	mas-linker-QC-miniG-AS	CGCCCTTGCTCACTCCACCAAGCTTGATTCCCTTGCTCTT GCTACTCCCCATGGTGGCGAC
16	FYVE-Venus-miniG-short-AS	AGCTCGAGATCTGAGTC
17	FYVE-Venus-miniG-long-AS	GATTCCACCTCCAGCTCGAGATCTGAGTC
18	S684-S-NheI-FYVE	tattgctagcGCCGCCATGTACCCATACGACGTCC
19	FYVE-mVenus-S	GCATCAGGAGGAAAGCTTgtgagcaagggcg
20	FYVE-mG AS	cgcccttgctcacAAGCTTTCCTCCTGATGC
21	miniG-XhoI-S	AGATCTCGAGCTGGA
22	Gsi43-AS-EcoRI	gaattcctGAAAAGCCCGCAATC
23	miniG-XhoI-S	AGATCTCGAGCTGGAGG
24	Gsq71-AS-EcoRI	gaattccaCACCAAATTATATTCCCGCAAGTT
25	S877/S878-S-NheI	CAGATCCGCTAGCGC
26	S877-noStop-Hind-AS	TGACTGAAGCTTAAAACCCGCAATCCC
27	S878-noStop-Hind-AS	TGACTCAAGCTTCACCAAATTATATTCCCGCAAGTT
28	NheI-Kozak-ATG-ARRB2-S	TTCgctagcGCCACCatgggggagaa
29	KpnI-no-Stop-ARRB2-AS	CGCGGTACCggCAGTGCCTCGACT
30	EcoRI-Kozak-ATG-ratCHRM3-S	TTCgaattcGCCACCatgaccttgcacagtaacag
31	KpnI-no-Stop-ratCHRM3-AS	CGCGGTACCggcaaggcctgctccg
32	NheI-Kozak-ATG-mY2R-S	TTCgctagcGCCACCatgGGCCCGGTAGG
33	EcoRI-no-Stop-mY2R-AS	TGCAGAATTCgCACATTGGTAGCCTC
34	mNPY2R-S	CCTACGACGTCCCCGACTACGCC
35	mNPY2R-AS	CTTATCGTCATCGTCCTTATAATC
36	mNPY2R	CCTACGACGTCCCCGACTACGCCGGCCCGGTAGGTGCAG AGGC
37	mNPY2R	GATCCACTAGTTCACTTATCGTCATCGTCCTTATAATCCA CATTGGTAGCCTCCGAAAAAGA
38	ACTB-1412-S	ACAATGTGGCCGAGGACTTT
39	ACTB-1519-AS	TGGGGTGGCTTTTAGGATGG
40	RPS18-106-S	GATGGGCGGCGGAAAATAG
41	RPS18-192-AS	GTCTGCTTTCCTCAACACCAC
42	SLC16A1-1501-S	GACCTTGTTGGACCCCAGAG
43	SLC16A1-1598-AS	AGCCGACCTAAAAGTGGTGG
44	SLC16A3-1038-S	TCCATGTTCTTCAACGGCCT
45	SLC16A3-1137-AS	TGCCGTAGGAGATGCCAAAG
46	SLC16A4-829-S	GCAGCAGTTTGTCTGCACAT
47	SLC16A4-932-AS	TCCAGCCTTCTGCGTAGTAC
48	SLC16A7-262-S	GACTCTGGGACTCTTGGTGC
49	SLC16A7-334-AS	GGTGGCATTTCTGCTCCTCT
50	SLC16A8-359-S	ATCCTCGTGACCCGCTTTG
51	SLC16A8-448-AS	CGTGGCAAAGGAAGCTAGGA
52	SLC13A4-2246-S	TTGTGAGCAACCCAGCAAC
53	SLC13A4-2336-AS	ATCAGGGTGTAGAGGGGGTT

54	TNFa-1165-S	CAGGGAGCCTTTGGTTCTGG
55	TNFa-1286-AS	CCGTGTCTCAAGGAAGTCTGG
56	IL-1b-915-S	GAGTCCTGTGCTGAATGTGGA
57	IL-1b-1029-AS	GCAGTTGGGCATTGGTGTAG
58	CXCL10-361-S	CTGAAAGCAGTTAGCAAGGAAAGG
59	CXCL10-481-AS	TGTAGGGAAGTGATGGGAGAGG
60	HLADRA-886-S	GCAGTCATCTTCAGCGTTTTCC
61	HLADRA-997-AS	AGGAAAAGGCAATAGACAGGGAAG
62	FN1-5972-S	GGAGAATGTCAGCCCACCAA
63	FN1-6095-AS	ATTGGCTGGAACGGCATCAA
64	IL10-1012-S	TTATCTTGTCTCTGGGCTTGGG
65	IL10-1148-AS	GAAGTGGTTGGGGAATGAGGT
66	CCL18-447-S	GAGTCCCATCTGCTATGCCC
67	CCL18-610-AS	TGAAGGGAAAGGGGAAAGGATG
68	CCL22-640-S	TCTGGGTTCCATCTCTGTCTCC
69	CCL22-757-AS	AACATCCCAGGCAAAGACCC

References

1. Rabe P, Liebing A-D, Krumbholz P, Kraft R & Stäubert C (2022). Succinate receptor 1 inhibits mitochondrial respiration in cancer cells addicted to glutamine. *Cancer letters* **526,** 91–102.
2. Peters A, Rabe P, Krumbholz P, Kalwa H, Kraft R, Schöneberg T & Stäubert C (2020). Natural biased signaling of hydroxycarboxylic acid receptor 3 and G protein-coupled receptor 84. *Cell Commun Signal* **18,** 31.
3. Lan T-H, Liu Q, Li C, Wu G & Lambert NA (2012). Sensitive and high resolution localization and tracking of membrane proteins in live cells with BRET. *Traffic* **13,** 1450–1456.
4. Maziarz M, Park J-C, Leyme A, Marivin A, Garcia-Lopez A, Patel PP & Garcia-Marcos M (2020). Revealing the Activity of Trimeric G-proteins in Live Cells with a Versatile Biosensor Design. *Cell* **182,** 770-785.e16.
5. Masuho I, Ostrovskaya O, Kramer GM, Jones CD, Xie K & Martemyanov KA (2015). Distinct profiles of functional discrimination among G proteins determine the actions of G protein–coupled receptors. *Science signaling* **8,** ra123.
6. Wan Q, Okashah N, Inoue A, Nehmé R, Carpenter B, Tate CG & Lambert NA (2018). Mini G protein probes for active G protein-coupled receptors (GPCRs) in live cells. *The Journal of biological chemistry* **293,** 7466–7473.
7. Weiss TS, Chamberlain CE, Takeda T, Lin P, Hahn KM & Farquhar MG (2001). Galpha i3 binding to calnuc on Golgi membranes in living cells monitored by fluorescence resonance energy transfer of green fluorescent protein fusion proteins. *Proceedings of the National Academy of Sciences of the United States of America* **98,** 14961–14966.
8. Ghosh P, Garcia-Marcos M, Bornheimer SJ & Farquhar MG (2008). Activation of Galphai3 triggers cell migration via regulation of GIV. *The Journal of cell biology* **182,** 381–393.
9. Qin K, Dong C, Wu G & Lambert NA (2011). Inactive-state preassembly of G(q)-coupled receptors and G(q) heterotrimers. *Nature chemical biology* **7,** 740–747.
10. Marivin A, Leyme A, Parag-Sharma K, DiGiacomo V, Cheung AY, Nguyen LT, Dominguez I & Garcia-Marcos M (2016). Dominant-negative Gα subunits are a mechanism of dysregulated heterotrimeric G protein signaling in human disease. *Science signaling* **9,** ra37.
11. Hollins B, Kuravi S, Digby GJ & Lambert NA (2009). The c-terminus of GRK3 indicates rapid dissociation of G protein heterotrimers. *Cellular signalling* **21,** 1015–1021.
12. Tóth JT, Gulyás G, Hunyady L & Várnai P. Development of Nonspecific BRET-Based Biosensors to Monitor Plasma Membrane Inositol Lipids in Living Cells. In *Intracellular Lipid Transport,* pp. 23–34. Humana Press, New York, NY.
13. Thor D, Schulz A, Hermsdorf T & Schöneberg T (2008). Generation of an agonistic binding site for blockers of the M(3) muscarinic acetylcholine receptor. *The Biochemical journal* **412,** 103–112.
14. Okayama H & Berg P (1983). A cDNA cloning vector that permits expression of cDNA inserts in mammalian cells. *Molecular and cellular biology* **3,** 280–289.
